# From Environmental Organism to Nosocomial Threat: *Serratia* spp. in the Era of Antimicrobial Resistance and Therapeutic Innovation

**DOI:** 10.3390/antibiotics15060575

**Published:** 2026-06-04

**Authors:** Ivana Cirkovic, Natalija Krca, Snezana Brkic

**Affiliations:** 1 Institute of Microbiology and Immunology, Faculty of Medicine, University of Belgrade, 11000 Belgrade, Serbia; natalijakrca@gmail.com; 2 Department of Medical Microbiology, University Clinical Center of Serbia, 11000 Belgrade, Serbia; brkic.snezana@gmail.com

**Keywords:** *Serratia* spp., healthcare-associated infections, multidrug-resistant, AmpC β-lactamase, carbapenemases, virulence factors, therapeutic strategies

## Abstract

*Serratia* spp., particularly *Serratia marcescens*, have emerged as clinically important opportunistic pathogens and are increasingly recognized as causes of healthcare-associated infections, especially among critically ill and immunocompromised patients. Their remarkable ecological adaptability, persistence in hospital environments, and capacity to acquire multiple antimicrobial resistance determinants have contributed to the global emergence of multidrug-resistant strains and complicated therapeutic management. This review aims to comprehensively analyze the epidemiology, virulence mechanisms, antimicrobial resistance patterns, and current and emerging therapeutic strategies associated with *Serratia* spp. The manuscript is based on a critical review and analysis of previously published literature retrieved from electronic scientific databases focusing on clinically relevant *Serratia* spp. infections and resistance trends. The reviewed literature demonstrates that *Serratia* spp. combine intrinsic resistance mechanisms, particularly inducible chromosomal AmpC β-lactamases, with acquired resistance determinants including extended-spectrum β-lactamases, carbapenemases, aminoglycoside-modifying enzymes, and plasmid-mediated quinolone resistance. Horizontal gene transfer and biofilm formation further enhance bacterial persistence, dissemination, and adaptation within healthcare settings. Clinically, these pathogens are associated with device-related infections, bloodstream infections, pneumonia, urinary tract infections, and hospital outbreaks, where increasing multidrug and carbapenem resistance significantly limits therapeutic options. Novel β-lactam/β-lactamase inhibitor combinations and cefiderocol represent promising therapeutic approaches, although treatment success remains highly dependent on accurate identification of underlying resistance mechanisms. This review highlights the growing public health importance of *Serratia* spp. and underscores the need for improved surveillance, molecular diagnostics, antimicrobial stewardship, and the development of innovative therapeutic strategies in the context of the evolving antimicrobial resistance crisis.

## 1. Introduction

Despite being historically overlooked and long regarded as a harmless environmental microorganism, the genus *Serratia*, particularly *Serratia marcescens*, has emerged as a clinically relevant opportunistic pathogen in the modern antimicrobial era [1,2]. *S. marcescens* was first described in 1819 by the Italian pharmacist Bartolomeo Bizio following the observation of a characteristic red pigmentation on food substrates such as polenta and rice [3]. Owing to this distinctive appearance, the organism was initially misclassified as a fungus, which contributed to a prolonged underestimation of its clinical significance [3,4]. This early misinterpretation, further reinforced by its association with so-called “bleeding” food phenomena, shaped its perception as a biologically insignificant organism for decades.

This underappreciation persisted well into the 20th century, when *S. marcescens* was utilized in environmental and military dispersion experiments, reflecting a prevailing belief in its biological innocuity [4]. However, subsequent clinical observations demonstrated its capacity to cause a wide spectrum of infections, including bloodstream infections (BSIs), pneumonia, urinary tract infections (UTIs), and endocarditis, particularly in hospitalized and immunocompromised individuals [2,5]. These findings prompted a critical reassessment of its role in human disease and marked its transition from a presumed saprophyte to a clinically important pathogen. In this context, *S. marcescens* exemplifies how environmental microorganisms can emerge as significant opportunistic pathogens in settings characterized by intensive medical interventions and increased host susceptibility.

The genus *Serratia* comprises a group of ubiquitous microorganisms widely distributed across diverse environmental niches, including water, soil, plants, animals, and hospital environments [1]. Among the recognized species, *S. marcescens* represents the most clinically significant and frequently isolated member and is therefore considered the primary representative of the genus in human pathology [1,2]. Its remarkable ecological versatility—together with its ability to persist on abiotic surfaces, tolerate adverse environmental conditions, and form biofilms on medical devices—facilitates long-term survival in healthcare settings and promotes both colonization and transmission [1,4]. These characteristics are particularly relevant in modern hospital environments, where invasive procedures and medical devices create favorable conditions for opportunistic pathogens.

From an epidemiological perspective, *Serratia* spp. have emerged as important contributors to healthcare-associated infections (HAIs), particularly in high-risk settings such as intensive care units (ICUs) and neonatal intensive care units (NICUs), where vulnerable populations, including preterm neonates and critically ill patients, are especially susceptible [4,6]. Surveillance data indicate that *Serratia* spp. rank among the frequently isolated Gram-negative pathogens in ICU settings, particularly in cases of pneumonia, BSIs and UTIs [4]. In clinical practice, *Serratia* spp. are most commonly isolated from respiratory tract specimens and blood cultures, reflecting their strong association with pneumonia and sepsis [6]. Longitudinal hospital-based studies report that respiratory samples account for approximately one-third of isolates, followed by BSIs (21.5%), further underscoring the role of *Serratia* spp. in severe invasive disease [6]. Moreover, the organism’s ability to persist under adverse conditions, combined with its capacity for clonal dissemination, further contributes to its epidemiological success and complicates infection control measures. Numerous outbreaks have been documented worldwide, frequently associated with contaminated medical equipment, environmental reservoirs, invasive devices, and transmission through the hands of healthcare workers [1,6].

In the context of escalating antimicrobial resistance (AMR), *Serratia* spp. pose an additional and increasingly significant challenge. *Serratia* spp. exhibit intrinsic resistance to multiple antibiotic classes while also possessing the capacity to acquire and disseminate diverse resistance mechanisms through horizontal gene transfer [1,5]. The presence of chromosomally encoded AmpC β-lactamases, along with the emergence of extended-spectrum β-lactamases (ESBLs) and carbapenemases, significantly limits therapeutic options and complicates clinical management. The group “ESCPM”, which contains *S. marcescens*, *Enterobacter* spp., *Citrobacter freundii*, *Providencia* spp., and *Morganella morganii*, exhibits elevated levels of AmpC expression, which contributes to an extensive resistance profile that includes various β-lactam antibiotics [1,4,7]. Furthermore, the continuous evolution of resistance profiles and the increasing prevalence of multidrug-resistant (MDR) strains underscore the urgent need for improved antimicrobial stewardship and the development of targeted therapeutic strategies.

The rationale for this review is therefore rooted in the need to re-evaluate the clinical importance of *Serratia* spp. in light of their increasing relevance in HAIs and AMR. Despite their significance, these organisms remain relatively underrepresented in the broader discourse on AMR compared to other Gram-negative pathogens, further highlighting the importance of focused investigation.

Accordingly, this review provides a comprehensive and integrative global overview of *Serratia* spp., with particular emphasis on *S. marcescens*, while also addressing region-specific epidemiological patterns, resistance mechanisms, and clinical challenges associated with these infections worldwide.

## 2. Taxonomy, Phenotypic Characteristics, and Ecology of *Serratia* spp.

### 2.1. Taxonomic Classification

The genus *Serratia* comprises a well-defined group of Gram-negative bacteria classified within the order *Enterobacterales* and the family *Yersiniaceae*. This taxonomic placement is supported by genome-based phylogenetic analyses that reorganized the former *Enterobacteriaceae* into several distinct families, including *Yersiniaceae* [8]. Subsequent whole-genome sequencing (WGS) studies, multilocus sequence typing (MLST) analysis, average nucleotide identity, and digital DNA–DNA hybridization have confirmed that *Serratia* spp. represent a coherent and phylogenetically distinct lineage within this family [9].

The genus *Serratia* currently consists of 24 validly published species worldwide, reflecting ongoing refinement of species boundaries through genome-resolved taxonomy [8,10,11,12]. These species have been isolated from a wide range of ecological niches, including soil, freshwater environments, plants, insects, animals, and clinical specimens, reflecting the ecological diversity and adaptability of the genus [8,9,10,11,12].

The genus was established with *S. marcescens* designated as the type species. *S. marcescens* is the most extensively studied representative of the genus due to its characteristic pigmentation and its importance as an opportunistic human pathogen. It has been frequently linked to HAIs, which have contributed significantly to its relevance in clinical microbiology and bacterial systematics [2]. Additionally, S. *marcescens* has been described as closely related to *Serratia nematodiphila*, *Serratia bockelmannii*, and *Serratia nevei* [13,14].

In addition to *S. marcescens*, several species are considered key representatives of the genus. *Serratia liquefaciens* is primarily associated with environmental habitats such as water and food products, although it has occasionally been isolated from clinical material. *Serratia rubidaea* is another pigment-producing species that has been recovered from both environmental and human sources, but it is reported far less frequently than *S. marcescens*.

**Figure 1:**
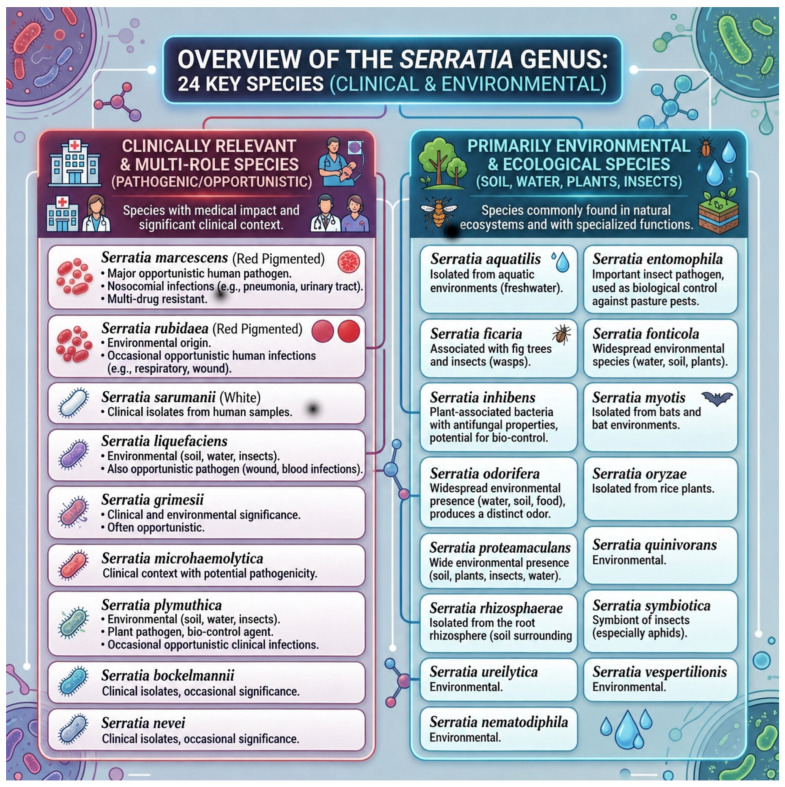
Diversity and key representative species of the genus *Serratia*. (Figure created in https://imgcreatorai.io/, accessed on 3 April 2026).

Other recognized species within the genus include *Serratia aquatilis*, *Serratia entomophila*, *Serratia ficaria*, *Serratia fonticola*, *Serratia grimesii*, *Serratia inhibens*, *Serratia microhaemolytica*, *Serratia myotis*, *Serratia odorifera*, *Serratia oryzae*, *Serratia plymuthica*, *Serratia proteamaculans*, *Serratia quinivorans*, *Serratia rhizosphaerae*, *Serratia sarumanii*, *Serratia symbiotica*, *Serratia ureilytica* and *Serratia vespertilionis* (Figure 1) [15]. The most recently described member of the genus, *S. sarumanii*, was formally proposed in 2024 based on comprehensive phenotypic and genomic characterization of clinical isolates and is distinguished by its white-pigmented phenotype, in contrast to the red-pigmented *S. marcescens* [12].

Accurate taxonomic classification of *Serratia* spp. is essential for reliable identification in clinical diagnostics, for epidemiological surveillance, and for understanding the ecological and biotechnological significance of this genus. Continuous advances in genome-based taxonomy are expected to further refine species boundaries and phylogenetic relationships within *Serratia* spp.

### 2.2. Phenotypic Characteristics and Virulence Factors of Serratia *spp*.

Members of the genus *Serratia* are Gram-negative, facultatively anaerobic bacilli [16]. Cells typically measure 0.5–0.8 μm in width and 0.9–2.0 μm in length and are motile by means of peritrichous flagella [2]. They are oxidase-negative, catalase-positive, capable of fermenting glucose, and reduce nitrate to nitrite. They grow readily on routine laboratory media, including blood agar, chocolate agar, and MacConkey agar, forming relatively large colonies after overnight incubation. Optimal growth generally occurs between 30 and 37 °C [2,16].

Differentiation of *Serratia* spp. may involve additional biochemical testing, including ornithine decarboxylase activity and carbohydrate fermentation profiles (e.g., arabinose, raffinose), as described in classical identification schemes [2]. *S. marcescens* is typically characterized by the frequent production of DNase, lipase, and gelatinase, a combination that distinguishes it from many other enterobacterial species in clinical microbiology [2]. However, due to phenotypic variability, modern clinical laboratories increasingly rely on matrix-assisted laser desorption/ionization time-of-flight mass spectrometry (MALDI-TOF MS) or molecular methods for precise species-level identification [2,17].

A hallmark phenotypic feature of several *Serratia* spp., particularly *S. marcescens* and *S. rubidaea*, is the production of the red tripyrrole pigment prodigiosin [2,16]. Pigment production is thermoregulated, with maximal expression between 25 and 30 °C and frequent suppression at 37 °C, which explains why many clinical isolates are non-pigmented [2,16,18]. Prodigiosin biosynthesis is under quorum-sensing (QS) control and has been associated with antimicrobial, immunomodulatory, and cytotoxic properties, although it is not essential for virulence in human infections [2,16,18,19,20].

The pathogenicity of *Serratia* spp. is mediated by a coordinated network of virulence determinants that promote colonization, persistence, immune evasion, and survival in both environmental and clinical settings. These mechanisms include adhesion factors, biofilm formation, secretion systems, extracellular enzymes, outer membrane proteins, and QS pathways. Importantly, several virulence-associated traits are closely interconnected with antimicrobial resistance and contribute to the persistence of multidrug-resistant strains in healthcare environments. The major virulence determinants of *Serratia* spp. are summarized in Table 1.

### 2.3. Environmental, Animal, and Healthcare-Associated Reservoirs of Serratia *spp*.

Members of the genus *Serratia* are widely distributed in nature and occupy diverse ecological niches [2,105,106,107].

Aquatic environments represent one of the primary natural habitats for *Serratia* spp. Several species, including *S. marcescens*, *S. fonticola*, *S. grimesii*, *S. liquefaciens*, *S. plymuthica*, *S. rubidaea*, and *S. ureilytica*, have been isolated from freshwater systems such as rivers and reservoirs, as well as from wastewater treatment systems [2,108]. In environmental surveys, *S. marcescens* and *S. liquefaciens* frequently predominate among *Serratia* spp. isolates recovered from river water [106]. These findings indicate that aquatic environments serve as important reservoirs that facilitate environmental persistence and potential dissemination.

Soil is another significant ecological niche for *Serratia* spp. Environmental isolates of *S. marcescens*, *S. grimesii*, *S. liquefaciens*, and *S. quinivorans* have been detected in various soil types, highlighting the adaptability of these bacteria to terrestrial ecosystems [109,110]. Because soil microbes are often dispersed through environmental disturbances, soil may represent an important source of contamination of food products, plants, and other environmental surfaces.

Many *Serratia* spp. are closely associated with plants and the plant rhizosphere. *S. marcescens* and *S. liquefaciens* have been isolated from a wide variety of plant species, including grasses, tomatoes, and green onions [110]. Some species exhibit plant-beneficial properties, as *S. plymuthica* has been shown to promote plant growth and suppress soilborne plant pathogens, supporting its classification as a plant growth-promoting rhizobacterium [2,111]. Similarly, *S. liquefaciens*, *S. plymuthica*, and *S. rubidaea* have been identified in the rhizosphere of oilseed rape and have demonstrated antifungal activity against plant pathogens [110]. In rare cases, however, members of the genus may also act as phytopathogens, as observed for *S. proteamaculans*, which causes leaf spot disease in *Protea cynaroides* [106].

Associations between *Serratia* spp. and animals have also been widely documented. *S. marcescens* has been isolated from numerous animal hosts, including reptiles, birds, rodents, pigs, goats, horses, and fish [2]. In poultry, *S. marcescens* may colonize the digestive tract of hens, leading to contamination of eggs and, in some cases, embryonic mortality, while *S. liquefaciens* has been detected on contaminated chicken carcasses [106]. In cattle and cows, *Serratia* spp. are associated with chronic mastitis, contributing to bacterial contamination of milk and dairy products, occasionally manifested as red discoloration caused by pigmented strains [106]. *S. marcescens* has also been implicated in coral disease, including white pox disease affecting the Caribbean elkhorn coral (*Acropora palmata*) [112].

Numerous investigations have demonstrated the presence of *Serratia* spp. isolates resistant to extended-spectrum cephalosporins in dogs and cats, as well as multidrug-resistant strains in farm animals and poultry [113]. These findings suggest that domestic animals may contribute to the maintenance, amplification, and potential dissemination of antimicrobial-resistant *Serratia* spp. strains within veterinary, agricultural, and possibly human-associated environments.

Insects also represent important ecological hosts for *Serratia* spp. Several species, including *S. marcescens*, *S. plymuthica*, *S. liquefaciens*, and *S. ficaria*, have been isolated from the natural microbiota of insects such as flies, termites, wasps, and grasshoppers [106]. Some *Serratia* spp. exhibit entomopathogenic activity; for instance, *S. entomophila* and *S. proteamaculans* cause amber disease in grass grubs, while *S. marcescens* has been reported to infect more than 70 insect species [114]. These interactions suggest that insects may act both as reservoirs and vectors, facilitating environmental dissemination.

Although *Serratia* spp. are naturally environmental organisms, they are also well adapted to survive in anthropogenic environments, particularly hospital settings. Hospital environments provide numerous ecological niches that support the persistence of *S. marcescens*, particularly in areas with frequent water exposure or inadequate disinfection practices. Water systems, sinks, faucets, and other moist surfaces are recognized as important reservoirs for opportunistic Gram-negative bacteria in healthcare facilities [115].

Recent studies have identified ICU environments, particularly sinks and drainage systems, as reservoirs of *Serratia* spp., where persistent clones can remain for over a year and are often genetically related to clinical isolates. These findings support a “source–sink” model, in which hospital plumbing serves as a long-term source of bacteria, while patients act as transient sinks for colonization and infection [116,117].

Transmission pathways in healthcare settings are often complex, involving multiple environmental and human reservoirs, including contaminated medical devices, colonized patients, inadequately disinfected or intrinsically contaminated solutions, and the hands of healthcare workers, that together can sustain bacterial survival and facilitate intra-facility spread [118].

These environmental reservoirs promote bacterial colonization and enable prolonged persistence within hospital infrastructure. The survival of *Serratia* spp. in such settings is supported by several virulence and adaptation mechanisms, including adhesion factors and flagella-mediated motility that enable attachment to abiotic surfaces and water systems. In addition, the capacity to form biofilms enhances resistance to disinfectants and environmental stress, thereby promoting persistence in moist hospital niches and increasing the likelihood of bacterial dissemination within healthcare environments [115,118].

**Figure 2:**
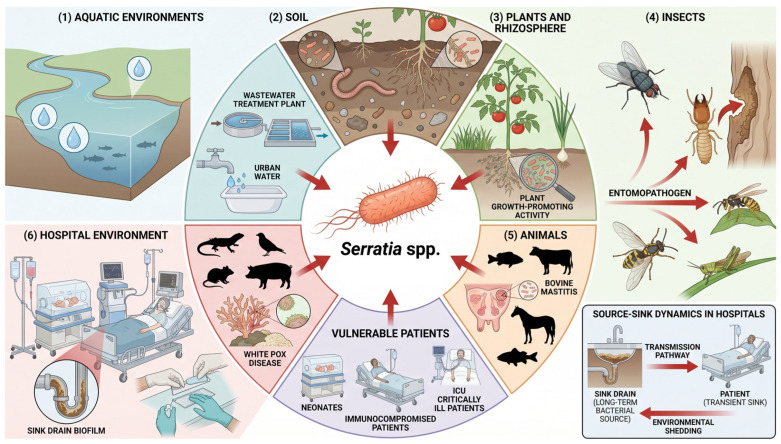
Ecological reservoirs and environmental distribution of *Serratia* spp. across natural and healthcare settings. (Figure created in https://imgcreatorai.io/, accessed on 27 April 2026).

Overall, the ecological versatility of *Serratia* spp. allows them to persist in a wide range of natural and artificial reservoirs (Figure 2). This environmental ubiquity, combined with intrinsic resistance traits and virulence factors, enables certain species, particularly *S. marcescens*, to act as opportunistic pathogens and emerge as important agents of HAIs.

## 3. Clinical Significance of *Serratia* spp.

From a clinical perspective, *S. marcescens* represents the most frequently isolated species within the genus and is responsible for the majority of *Serratia*-associated infections in healthcare settings, playing a central role in hospital outbreaks. In addition to *S. marcescens*, several other species, including *S. liquefaciens*, *S. ficaria*, *S. fonticola*, *S. grimesii*, *S. rubidaea*, and *S. plymuthica*, have increasingly been recognized as clinically relevant pathogens implicated in a variety of human infections [2,119].

*Serratia* spp. are associated with a wide range of infections, including pneumonia, UTIs, sepsis, wound and device-associated infections, meningitis, and ocular infections, among others [2]. According to the European Centre for Disease Prevention and Control (ECDC), *Serratia* spp. account for approximately 6.4% of pneumonia cases in the ICU, 3.9% of ICU-acquired BSIs, 1.4% of UTIs in the ICU, and 1.2% of surgical site infections (SSIs) in Europe [120,121].

The earliest probable report of human infection with *S. marcescens* dates to 1913, when a red-pigmented organism, then termed *Bacterium prodigiosum*, was isolated from the sputum of a patient with chronic cough [2]. In addition to *S. marcescens*, other species, including *S. liquefaciens*, *S. ficaria*, *S. rubidaea*, and the recently described *S. sarumanii*, have also been implicated in respiratory infections [2,122]. Due to their relatively low virulence, *Serratia* spp. rarely cause respiratory infections in healthy individuals. However, they represent an important cause of hospital-acquired and ventilator-associated pneumonia, particularly in ICU patients. These infections are commonly associated with mechanical ventilation, prolonged hospitalization, and prior exposure to antibiotics [123]. Antibiotic pressure, whether prophylactic or therapeutic, facilitates colonization by *Serratia* spp., reflecting their substantial repertoire of AMR determinants, including intrinsic mechanisms (e.g., AmpC-type β-lactamases) and acquired genes such as *bla*_CTX-M_ or carbapenemases [122,123].

BSIs caused by *Serratia* spp. are strongly associated with intravascular devices and invasive procedures. Virulence factors, such as a structurally variable polysaccharide capsule, impair immune clearance, enabling bloodstream survival, device colonization, and persistent bacteremia [119]. Although *S. marcescens* remains the predominant species, non-marcescens isolates, particularly *S. liquefaciens* and *S. odorifera*, are being reported with increasing frequency in cases of nosocomial bacteremia [2,119]. Besides cardiac and endovascular devices, SSIs, chronic wounds, and contaminated percutaneous devices are documented sources of nosocomial *Serratia* spp. bacteremia, especially in patients in the ICU [119]. Beyond critically ill patients, intravenous drug users also represent a high-risk group due to non-sterile injection practices, use of tap or environmental water for drug dilution, and frequent healthcare exposure [119].

*Serratia* spp. are recognized causes of healthcare-associated UTIs, predominantly linked to urinary catheterization and prior antimicrobial exposure. While *S. marcescens* is the principal pathogen, *S. liquefaciens* and *S. fonticola* have been reported in complicated UTIs, particularly in immunocompromised or critically ill patients [2]. The study of Moreno et al. demonstrated a significantly higher prevalence of *S. marcescens*-complicated UTIs in catheterized vs. non-catheterized patients [124]. Catheterization was also linked to higher rates of recurrent UTIs, recent antibiotic use (within the past three months), and 30-day unplanned hospital readmissions, contributing to increased AMR in *Serratia* spp. isolates and further limiting treatment options [124].

*Serratia* spp. represent important etiological agents of wound- and device-associated infections, particularly due to their propensity to colonize moist niches in burn wounds and to form biofilms on medical devices. SSIs, catheter-related infections, and infections involving implanted devices have been reported not only for *S. marcescens* but also for *S. liquefaciens* and *S. plymuthica*, often resulting in persistent or recurrent clinical courses [2,125]. Posluszny et al. documented that *S. marcescens* accounted for 11% of burn-related SSIs, with significant associations with autografting, regrafting procedures, and prolonged hospitalization [126]. Furthermore, *S. marcescens* has been implicated in necrotizing fasciitis, a severe and rapidly progressive soft-tissue infection associated with mortality rates of 24% or higher [127].

*S. marcescens* is an opportunistic pathogen responsible for a wide range of ocular infections, including conjunctivitis, keratitis, keratoconjunctivitis, corneal ulcers, and endophthalmitis, in both previously healthy and injured eyes, particularly in hospital settings and among contact lens users. Its environmental ubiquity supports ocular colonization. Other *Serratia* spp. such as *S. liquefaciens*, *S. plymuthica*, *S. ficaria*, and *S. rubidaea* have also been associated with contaminated contact lenses, corneal abscesses, and post-traumatic or burn-related eye infections [2]. In NICUs, *S. marcescens* is a notable cause of bacterial conjunctivitis associated with prolonged hospitalization and supportive care, where purulent discharge may indicate late-onset sepsis, occasionally involving *S. marcescens* in systemic infections [128].

Among the earliest documented clinical infections of *Serratia* spp. was a report from 1942, describing meningitis in a United States (US) Army soldier in whom red-pigmented Gram-negative bacteria identified as *S. marcescens* were isolated from cerebrospinal fluid [2]. Central nervous system (CNS) infections caused by *Serratia* spp. are rare but severe, occurring mainly in neonates or profoundly immunocompromised patients. In adults, a history of head trauma, neurosurgical procedures, mastoiditis or chronic sinusitis is associated with CNS infections [129]. Neonatal meningitis and ventriculitis are predominantly attributed to *S. marcescens*, although sporadic cases involving other species have been reported [2].

*Serratia* spp. are opportunistic nosocomial pathogens with low inherent virulence, but they have a substantial clinical impact in vulnerable hosts. The highest risk is observed in immunocompromised patients (malignancy, transplantation, neutropenia, and immunosuppressive therapy) and critically ill ICU patients exposed to mechanical ventilation, central venous catheters, broad-spectrum antibiotics, and prolonged hospitalization [2,119,122,123]. Preterm and very-low-birth-weight neonates represent a high-risk group. *Serratia* spp., especially *S. marcescens*, are well-recognized causes of late-onset sepsis and outbreaks in NICUs, often linked to environmental reservoirs or contaminated equipment [2,128,130,131]. Additional risk is conferred by indwelling devices (central lines, urinary catheters, and endotracheal tubes) due to biofilm formation [2,122,123,124,130,131]. Disruption of skin barriers, extensive tissue damage, and prolonged ICU care predispose burn and trauma patients to opportunistic infections caused by *Serratia* spp., including wound infections and secondary bacteremia [125,126,127].

Disease severity of *Serratia* spp. infections depends largely on the infection site, with BSIs associated with substantial morbidity and mortality, with mortality reaching ~30% or higher in high-risk populations with extensive comorbidities or drug resistance. Device-associated bacteremia increases the likelihood of persistent infection and poor outcomes [119]. Neonatal *S. marcescens* sepsis carries high case fatality (~32–58%), particularly with invasive support or concomitant meningitis [2,128,130,131], although mortality in *S. marcescens* meningitis may be lower than in other Gram-negative etiologies [129]. Prior antibiotic exposure and the emergence of MDR or carbapenem-resistant (CR) strains further complicate therapy, contributing to prolonged hospitalization and increased mortality [123,124,129,130,131].

## 4. Hospital Outbreaks and Infection Control

Documented outbreaks of *Serratia* spp. illustrate a consistent pattern of opportunistic, healthcare-associated transmission, often linked to moist environmental niches and contaminated solutions or devices, facilitated by lapses in aseptic technique and hand hygiene.

Nosocomial outbreaks of *S. marcescens* have been extensively documented in adult populations since the late 1960s, affecting a wide range of hospital settings, including ICUs, surgical wards, transplant units, and outpatient facilities, with some outbreaks spanning multiple wards, hospitals, or even regions [2]. Notable multistate outbreaks include BSIs linked to contaminated intravenous magnesium sulfate in 2005 and prefilled heparin syringes in 2007–2008, the latter causing 162 infections and four deaths across nine US states, with 84% of *S. marcescens* isolates being genetically related [2,132]. Additional cases were associated with contaminated multidose heparin vials in China, where the same strain of *S. marcescens*, confirmed by pulsed-field gel electrophoresis (PFGE) profile, was isolated from blood samples of nine patients [133].

Recent reports extend this pattern. A cardiothoracic surgery ward outbreak was traced to contaminated pre-prepared heparin-flushing syringes, with 23 of 47 postoperative patients developing positive blood cultures for *S. marcescens* [134]. Furthermore, a large postsurgical bacteremia cluster was linked to fentanyl-containing intravenous pain-control fluids. In this outbreak, the same *S. marcescens* pulsotype was identified in 82.7% of positive blood cultures [135]. Non-sterile parenteral nutrition products have also been implicated as sources of outbreaks. Gupta et al. described an outbreak across six healthcare facilities in the US involving 19 patients with *S. marcescens* BSIs [136]. Another outbreak of severe *S. marcescens* bacteremia (eight cases, five deaths) was linked to strains resistant or tolerant to a quaternary ammonium disinfectant used in the ICU, enabling environmental persistence [118,137].

Contaminated equipment is a significant source of outbreak-associated bacteria, ranging from endoscopes to razors and brushes. The latter was linked to clusters of craniotomy site infections in neurosurgery [138]. In the review by Kakoullis et al., *S. marcescens* was one of the main bacteria reported in bronchoscopy-related events. True outbreaks were uncommon, whereas pseudo-outbreaks predominated, with *S. marcescens*, often in mixed clusters alongside organisms like *Pseudomonas aeruginosa*, isolated from samples of patients who underwent bronchoscopy with contaminated instruments but did not develop pneumonia [139].

Other *Serratia* spp. are less frequently implicated in outbreaks compared to *S. marcescens*. *S. liquefaciens* has been linked to several hospital-acquired outbreaks, including infections among infants in an Australian neonatal unit (1976–1982) and neurosurgery patients in France (2005), where it was isolated from various clinical samples [2]. A nosocomial outbreak of *S. liquefaciens* UTIs was reported in patients undergoing cystometry, with 10 of 44 patients developing infection. The organism was traced to fluid within the dome connected to the pressure transducer used for pressure monitoring during the procedure [140]. The most notable incident occurred in 1999 at a Colorado hemodialysis center, where contaminated epoetin alfa led to multiple BSIs and pyrogenic reactions that ceased once contamination sources were removed [2]. In the national investigation in Norway, *S. ureilytica* (*S. marcescens* complex type 755) was identified across 33 hospitals (June 2021–February 2023), revealing a large, opportunistic, community-associated outbreak with no clear epidemiological links or confirmed common source, though a widely distributed product (possibly food) was suspected [141].

In neonates and pediatric patients, outbreaks have been reported since the 1950s, often with severe outcomes such as sepsis and high mortality in premature infants and linked to sources such as contaminated intravenous solutions, breast milk, medical equipment, and hospital environments, with conjunctivitis being particularly common in this population [2,128]. *S. marcescens* was responsible for a substantial number of neonatal outbreaks. A prolonged 18-month Belgian NICU outbreak affected 61 neonates (10 infections, including one fatal sepsis), with repeated “peaks” driven by different clonal clusters despite successive control rounds [142]. In Italy, a six-month outbreak involved 18 colonized or infected infants, mainly with ocular and rectal colonization [143], and in Mexico, a NICU cluster of 15 septic neonates occurred over two months, with one death but substantial morbidity and resource use [144]. Bechman et al. reported a NICU outbreak linked to donor breast milk contaminated with *S. marcescens* [145]. Maltezou et al. described three consecutive, rapidly spreading, and prolonged NICU outbreaks over a three-year period, during which 9 of 20 neonates with invasive *S. marcescens* infection died. Although the outbreak strains were genetically distinct, they were linked to a common environmental source—the milk-kitchen sink [146]. An outbreak of *S. marcescens* and *S. rubidaea* bacteremia occurred in the NICU of a Kathmandu hospital involving three severely ill, low-birth-weight premature neonates, one of whom died. The six blood culture isolates from these infants were genetically identical, suggesting a single-source outbreak likely linked to post-earthquake disruptions and lapses in infection control, although the exact source was not identified [147].

Across these reports, typical sources include contaminated intravenous drugs, flushing solutions (heparin–saline, fentanyl, and other analgesics), and parenteral nutrition products [2,132,133,134,135,136]; contaminated equipment (syringes, endoscopes, and catheters) [134,139,140]; contaminated water systems, water filters, sinks, and washbasin drains [139,142,146]; shaving equipment [138]; and occasionally disinfectants themselves [118,137]. *S. marcescens* is highly persistent in the environment, and its ability to form biofilms, including in disinfectant systems such as those using quaternary ammonium compounds, enables it to survive routine cleaning and generate “disinfectant-resistant” strains that can repeatedly reseed the wards [2,117,118,137,148]. Even incubator humidification water could sustain *Serratia* spp. despite regular disinfection, enabling reseeding over weeks to months [149].

In many *S. marcescens* nosocomial outbreaks, transmission pathways remain poorly defined, but person-to-person spread is repeatedly implicated, especially where workload is high, hand hygiene is imperfect, there are lapses in aseptic technique, or shared items (trolleys, razors, and multidose vials) are used across multiple patients or rooms [117,134,141,143,144,146,150]. Even handwashing sinks and drains and plain or liquid soap used at sinks could be contaminated via infected or colonized patients, consequently leading to colonization of medical equipment or the beds adjacent to the sink through splashing [117,149]. Infection prevention strategies in the outbreak reports consistently emphasize reinforced hand hygiene, targeted education of staff on the issues of hand hygiene and use of personal protective equipment, and direct observation with feedback in affected units [137,144,145,146,148,149].

Environmental cleaning should be intensified with higher-frequency surface disinfection, replacement of ineffective disinfectants, and, when indicated, use of chlorine solutions or hydrogen peroxide nebulization to decontaminate rooms and devices [134,137,139,142,146,148]. Device-specific instructions should be consistently followed, with staff regularly trained and provided access to clear cleaning and disinfection protocols [139,145]. Using disposable equipment whenever possible is strongly recommended as a key preventive strategy [138]. Control of water systems and wet reservoirs is crucial, including systematic sampling of sinks and drains; removal or redesign of washbasins, especially in NICU rooms; installation (and, if ineffective, removal) of heated drains; and strict separation of handwashing sinks from fluid preparation and device-storage areas [117,137,142,146,149].

Routine and outbreak-driven surveillance cultures (rectal and pharyngeal screening, conjunctival swabs, and targeted environmental sampling) are used to detect silent carriage, guide cohorting, and track the effect of interventions, with several authors recommending continuous NICU-specific surveillance and drain monitoring even outside recognized outbreaks [135,142,149]. As WGS analyses repeatedly demonstrate close genetic relatedness between isolates from colonized infants and those from sinks or donor milk, ongoing surveillance could allow early detection of silent ward transmission and help predict the outbreak potential of a genetically well-characterized pathogen [142,149]. A cautious approach is required when considering rectal screening of healthcare workers without signs of infection, as it offers low sensitivity, is costly, and causes discomfort. Instead, greater emphasis should be placed on examining their hands for unusual features (such as artificial nails, rings, or onychomycotic lesions) before initiating any broad screening of otherwise healthy staff [150].

Across these reports, outbreak detection was based on promptly recognizing unusual infection clusters, performing systematic clinical and environmental sampling, and, increasingly, applying whole-genome typing to confirm relatedness and optimize control strategies. To distinguish clonal outbreaks from unrelated sporadic cases, a range of methods, including serotyping and multiple molecular typing approaches (e.g., PFGE), has traditionally been used to demonstrate relatedness and trace sources such as contaminated drugs, devices, or disinfectants [2]. More recent studies have adopted WGS with core-genome MLST or single-nucleotide polymorphism-based phylogenies to delineate clusters, follow persistence, and separate multiple co-circulating clones [117,118,141,142,145,149].

**Figure 3:**
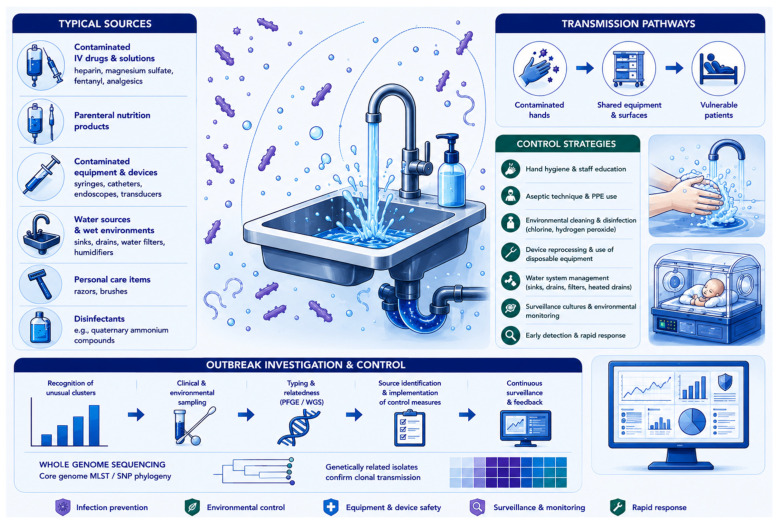
Sources, transmission pathways, outbreak investigation, and control measures in healthcare-associated *Serratia* spp. outbreaks. (Figure created in https://imgcreatorai.io/, accessed on 1 May 2026).

A schematic overview of the principal sources, transmission pathways, outbreak investigation approaches, and infection control interventions described in *Serratia* spp. outbreaks is presented in Figure 3.

Ultimately, effective control depends on rapid recognition of abnormal clustering, identification of a strong epidemiologic implication of a source, removal or replacement of contaminated materials, and consistent reinforcement of aseptic practices in high-risk procedures. In parallel, modern computer-assisted surveillance platforms that automatically merge microbiologic, administrative, and pharmacy data allow real-time or high-frequency detection of hospital-acquired outbreaks and subtle shifts in prevalence, thereby strengthening the early-warning function of microbiology laboratories and their close collaboration with infection control teams and markedly enhancing timely outbreak recognition and response [134].

## 5. Intrinsic Resistance

*Serratia* spp., particularly *S. marcescens*, are opportunistic Gram-negative pathogens characterized by a well-defined intrinsic resistance profile that significantly limits available therapeutic options. This intrinsic resistance is a fundamental property of the genus and reflects a combination of structural, biochemical, and regulatory mechanisms that reduce susceptibility to multiple classes of antimicrobial agents [2,105,106]. As members of the *Enterobacterales*, *Serratia* spp. possess a complex outer membrane that acts as a permeability barrier, as well as chromosomally encoded resistance determinants that contribute to their baseline antimicrobial phenotype.

**Figure 4:**
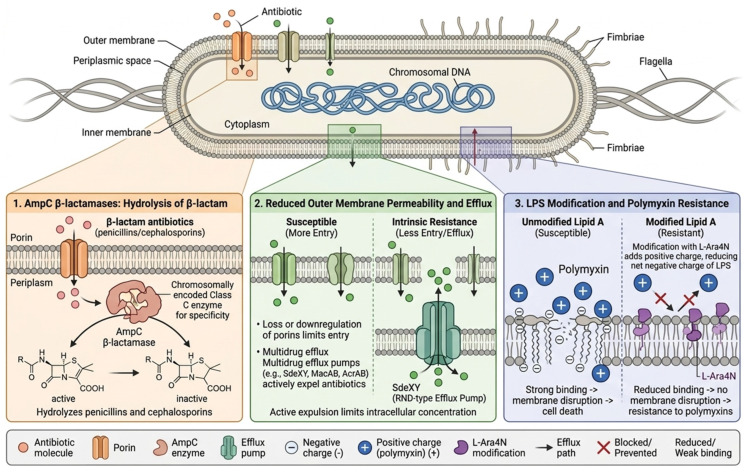
Intrinsic resistome and core AMR mechanisms in *Serratia* spp. (Figure created in https://imgcreatorai.io/, accessed on 1 May 2026).

Intrinsically, *Serratia* spp. are resistant to numerous antibiotics, including penicillin G, macrolides, clindamycin, linezolid, glycopeptides, quinupristin–dalfopristin, and rifampin [2,105,106]. In addition, most species within the genus exhibit natural resistance to aminopenicillins, β-lactam/β-lactamase inhibitor combinations, first- and second-generation cephalosporins, cephamycins, cefuroxime, nitrofurantoin, and polymyxins [2,151]. This broad intrinsic resistance profile is largely driven by the combined action of chromosomally encoded AmpC β-lactamases, reduced outer membrane permeability, active efflux systems, and lipid A modifications within LPS (Figure 4) [1,106,151].

These mechanisms do not act independently but rather synergistically, resulting in reduced intracellular accumulation of antibiotics and decreased target binding. The intrinsic resistome of *Serratia* spp. is therefore a key determinant of their survival in both environmental and clinical settings and provides a foundation upon which additional acquired resistance mechanisms may develop. Understanding these intrinsic factors is essential for appropriate antimicrobial selection and for predicting resistance evolution during therapy.

### 5.1. AmpC β-Lactamases

A central component of intrinsic resistance in *S. marcescens* is the expression of a chromosomally encoded AmpC β-lactamase, an Ambler class C cephalosporinase that contributes significantly to resistance against β-lactam antibiotics [151]. AmpC enzymes preferentially hydrolyze cephalosporins, including cephamycins, while also exhibiting activity against penicillins and monobactams such as aztreonam [151,152]. This enzymatic activity reduces susceptibility to a broad range of β-lactam agents and represents a defining feature of the intrinsic resistance phenotype in *Serratia* spp.

The *ampC* gene in *S. marcescens* is typically inducible and regulated through a conserved genetic network involving *ampR*, *ampD*, *ampG*, and *ampE*, which are functionally linked to peptidoglycan recycling pathways [152,153]. Under basal conditions, AmpC expression is relatively low; however, exposure to β-lactam antibiotics leads to the accumulation of cell wall degradation products in the cytoplasm. These muropeptides interact with the transcriptional regulator AmpR, converting it from a repressor into an activator of *ampC* transcription, thereby increasing β-lactamase production [152,153,154].

A distinctive feature of *S. marcescens* AmpC regulation is the presence of an extended 5′-untranslated region (~126 nucleotides) in the *ampC* transcript, which forms a stable stem–loop structure that enhances mRNA stability and may contribute to sustained enzyme expression [2]. This post-transcriptional regulatory mechanism differentiates *Serratia* spp. from other *Enterobacterales* and may influence the magnitude and duration of AmpC-mediated resistance.

Although AmpC production is inducible, mutations in regulatory genes, particularly *ampD*, can result in derepressed mutants characterized by constitutive high-level expression of AmpC β-lactamase [152,153]. These derepressed strains exhibit significantly increased minimum inhibitory concentrations (MICs) to multiple β-lactam antibiotics and are associated with clinical treatment failure, especially during therapy with third-generation cephalosporins [151,152].

Importantly, *S. marcescens* appears less prone to derepression compared with other AmpC-producing *Enterobacterales*, such as *Enterobacter cloacae* or *C. freundii*, with lower mutation frequencies reported in both experimental and clinical settings [1]. Nevertheless, the risk of selecting derepressed mutants during antimicrobial therapy remains clinically relevant, particularly when β-lactams that act as weak inducers but are susceptible to AmpC hydrolysis are used.

In addition to regulatory changes, structural mutations within the AmpC enzyme may expand its substrate spectrum. Amino acid substitutions in critical regions, such as the omega loop, can increase catalytic efficiency and confer resistance to extended-spectrum cephalosporins, including ceftazidime [151]. Similarly, alterations affecting structural elements of the enzyme have been associated with reduced susceptibility to fourth-generation cephalosporins such as cefepime [151]. Although such variants are relatively uncommon, they represent an important mechanism of adaptive resistance. Furthermore, AmpC-mediated resistance may be potentiated by additional mechanisms, including reduced outer membrane permeability [1].

Beyond *S. marcescens*, chromosomal AmpC β-lactamases are widely distributed among other *Serratia* spp., including *S. liquefaciens*, *S. plymuthica*, and *S. odorifera*, although differences exist in inducibility and expression levels [151]. This variability reflects evolutionary diversification within the genus and may influence species-specific resistance patterns.

### 5.2. Reduced Permeability and Efflux Pumps

Reduced outer membrane permeability and the activity of multidrug efflux pumps represent key complementary mechanisms of intrinsic resistance in *S. marcescens*. As a Gram-negative bacterium, *S. marcescens* possesses an outer membrane that acts as a selective permeability barrier, limiting the penetration of antimicrobial agents into the periplasmic space [155,156]. This barrier is largely mediated by LPS organization and porin composition, which together reduce passive diffusion of antibiotics into the periplasmic space.

Alterations in outer membrane permeability may further enhance resistance. For example, reduced expression or loss of porins, such as OmpF homologs, has been associated with decreased susceptibility to β-lactams and carbapenems, particularly when combined with AmpC overproduction [157]. This synergistic interaction highlights the importance of permeability defects as resistance amplifiers rather than standalone mechanisms.

In parallel, *S. marcescens* encodes a wide array of efflux systems that actively extrude toxic compounds, including antibiotics, from the bacterial cell. Genomic analyses have identified more than 70 efflux-associated genes distributed across clinical and environmental isolates, suggesting that these systems are an inherent part of the species’ resistome [1]. Efflux pumps contribute significantly to intrinsic resistance by lowering intracellular antibiotic concentrations below effective levels.

Multiple efflux pump families have been described in *S. marcescens*, including the resistance–nodulation–division (RND), major facilitator superfamily (MFS), small multidrug resistance (SMR), multidrug and toxic compound extrusion (MATE), and ATP-binding cassette (ABC) transporters [1]. Among these, RND-type efflux pumps are considered the most clinically relevant due to their broad substrate specificity and high transport capacity.

The SdeXY efflux system is one of the best-characterized RND pumps in *S. marcescens* and plays a central role in intrinsic multidrug resistance. Inactivation of *sdeXY* leads to increased susceptibility to multiple antibiotics, including tigecycline, fluoroquinolones, and certain β-lactams, indicating its major contribution to baseline resistance [1]. Similarly, the SdeAB efflux pump has been implicated in resistance to fluoroquinolones, chloramphenicol, and various biocides, although its activity appears to be strain-dependent [1].

Other RND systems, such as SdeCDE, SdeGH, and SdePQ-OmsA, contribute to resistance against specific substrates, including detergents, disinfectants, and bile salts, reflecting the ecological versatility of *S. marcescens* [1]. These pumps often function as tripartite complexes spanning the inner membrane, periplasm, and outer membrane, enabling direct extrusion of substrates into the external environment.

In addition to RND pumps, MFS transporters such as SmfY and TetA contribute to resistance against fluoroquinolones, tetracyclines, and cationic compounds, while SMR pumps like SsmE are involved in the extrusion of antiseptics and dyes [1]. Although individually these systems may confer modest resistance, their combined activity results in a robust MDR phenotype.

ABC-type efflux systems, including MacAB, also play a role in resistance, particularly against aminoglycosides and cationic antimicrobial peptides, and have been linked to additional physiological functions such as oxidative stress tolerance and biofilm formation [1]. This functional overlap suggests that efflux systems contribute not only to antibiotic resistance but also to bacterial survival and persistence in hostile environments.

### 5.3. LPS Modification (Polymyxin Resistance)

A major determinant of intrinsic resistance in *S. marcescens* is its inherent resistance to polymyxins, including colistin, which is mediated by structural modifications of LPS. Polymyxins exert their antibacterial effect by binding to the negatively charged phosphate groups of lipid A, disrupting the outer membrane, and leading to cell death [155,158]. However, *S. marcescens* is intrinsically resistant to these agents due to constitutive modification of lipid A, which reduces its net negative charge and consequently decreases the binding affinity of polymyxins [1,159].

The primary mechanism underlying this resistance is the addition of 4-amino-4-deoxy-L-arabinose (L-Ara4N) to lipid A and, in some cases, to the core oligosaccharide. This modification neutralizes the negative charge of LPS and significantly impairs electrostatic interactions with cationic antimicrobial peptides [1,159]. The process is mediated by the *arnBCADTEF* operon (also referred to as the *pmrHFIJKLM* operon), which encodes enzymes responsible for the biosynthesis and transfer of L-Ara4N to lipid A [1,159,160].

Regulation of LPS modification in *S. marcescens* is primarily controlled by the PhoP/PhoQ two-component regulatory system. Environmental signals such as low Mg^2+^ concentrations, acidic pH, or the presence of antimicrobial peptides activate the sensor kinase PhoQ, leading to phosphorylation of the response regulator PhoP, which in turn induces expression of the *arn* operon [159,161]. In addition, the PmrA/PmrB system can further enhance LPS modification through cross-regulation mediated by the connector protein PmrD, forming a coordinated regulatory network that modulates resistance [159,162].

Unlike many other *Enterobacterales*, in which polymyxin resistance is typically acquired, *S. marcescens* exhibits constitutive expression of LPS-modifying pathways, making polymyxin resistance an intrinsic trait of the species [1,159]. Experimental disruption of genes within the *arn* operon results in a marked increase in polymyxin susceptibility, confirming the essential role of this pathway in resistance [159]. Furthermore, regulatory elements such as MgrB may influence PhoP/PhoQ signaling and thereby modulate the extent of LPS modification [162].

In addition to L-Ara4N modification, other structural adaptations of LPS, including changes in acylation patterns of lipid A, may further contribute to reduced membrane permeability and resistance to host-derived antimicrobial peptides [158,161]. These changes not only impart antibiotic resistance but also improve bacterial survival under adverse conditions, including within the host during infection.

## 6. Acquired Resistance Mechanisms

### 6.1. Extended-Spectrum β-Lactamases

ESBLs represent an important mechanism of acquired resistance among *Enterobacterales*, including *Serratia* spp. These enzymes emerged following the introduction of broad-spectrum cephalosporins in the early 1980s, which were initially effective against organisms producing classical β-lactamases such as TEM and SHV [163]. ESBLs are typically plasmid-mediated enzymes capable of hydrolyzing penicillins; narrow-, expanded-, and broad-spectrum cephalosporins; and aztreonam [163]. The major ESBL families include TEM, SHV, OXA, and CTX-M enzymes, with CTX-M types now recognized as the most widespread globally. Indeed, CTX-M enzymes have largely replaced TEM and SHV variants in many regions and are currently the dominant ESBL type worldwide [164].

Although *S. marcescens* is not traditionally considered a primary ESBL producer compared to *Klebsiella pneumoniae* or *E. coli*, multiple studies have documented ESBL-producing strains, including those responsible for nosocomial outbreaks [165,166,167,168,169]. Among these, CTX-M-type enzymes predominate in *S. marcescens* isolates, although SHV and TEM variants have also been reported [2]. Additionally, rare ESBLs such as BES-1 have been described [170], highlighting the genetic diversity of resistance mechanisms in this species.

Beyond *S. marcescens*, other *Serratia* spp. may also act as reservoirs of ESBL genes. For example, ESBL-producing *S. fonticola* isolates carrying FONA-type β-lactamases (e.g., FONA-5) have been identified in environmental sources such as fresh vegetables, demonstrating the presence of less common ESBL variants within the genus [171]. These findings suggest that *Serratia* spp. may contribute to the environmental resistome and serve as a potential source of transferable resistance determinants.

The prevalence of ESBL-producing *S. marcescens* varies significantly across geographic regions. In Taiwan, studies conducted between 2001 and 2005 reported ESBL prevalence rates ranging from 12.2% to 16%, with CTX-M-3 identified as the dominant enzyme and associated with high mortality rates [172,173]. In South Korea, prevalence rates ranged from 12.4% to 30.6% [174,175], while in Thailand, 24.1% of isolates produced ESBLs, often harboring a combination of CTX-M, SHV, and TEM enzymes [176]. Similarly, studies from Mexico reported ESBL production in 20.5% of isolates, predominantly SHV-type enzymes (143), whereas in India, ESBL prevalence among *Serratia* spp. reached 33%, although species-level differentiation and molecular characterization were limited [177].

European data also demonstrate substantial variability. In Poland, ESBL production among *S. marcescens* isolates ranged from 19% in earlier hospital-based studies [168] to alarmingly high levels of 70.8% in a nationwide survey [178]. In this study, CTX-M enzymes accounted for the majority (80.1%) of ESBLs, with the remainder being SHV-type enzymes [178]. Furthermore, a study from a transplantation unit reported ESBL prevalence as high as 77.8%, compared to 26.3% in other hospital wards [179], emphasizing the impact of hospital setting and patient population on resistance rates.

Recent data also confirm that ESBL-producing Gram-negative bacilli remain a major global health problem, with high prevalence reported in certain settings. For example, a study in pediatric hospitals in Gaza reported an overall ESBL prevalence of 51.6% among Gram-negative isolates, although *S. marcescens* represented only a small proportion of isolates, indicating that while *Serratia* spp. are less frequent ESBL producers, they still contribute to the broader resistance landscape [164].

More recent data indicate that CTX-M enzymes continue to dominate globally among ESBL-producing *Enterobacterales*, including *Serratia* spp., driven by the spread of mobile genetic elements such as plasmids and transposons [180]. Surveillance data from Europe show increasing ESBL prevalence, particularly in Southern and Eastern regions, although *Serratia* spp. remain less frequently reported compared to *E. coli* and *K. pneumoniae*. Nevertheless, outbreaks involving ESBL-producing *S. marcescens* continue to be described, especially in ICUs and neonatal settings.

### 6.2. Carbapenemases

Carbapenem resistance in *Serratia* spp., particularly in *S. marcescens*, has evolved from a relatively uncommon phenomenon into a clinically relevant global problem driven largely by the acquisition and dissemination of carbapenemase genes. Historically, the earliest CR *S. marcescens* isolates were described in 1982, whereas the first carbapenemase gene sequenced from this species was *bla*_SME-1_ in 1994, establishing *Serratia* spp. as one of the earliest *Enterobacterales* in which a carbapenemase was molecularly characterized [181,182].

Since then, the carbapenemase repertoire of *S. marcescens* has expanded substantially and now includes chromosomal SME enzymes as well as a broad range of acquired plasmid-associated carbapenemases, including KPC, NDM, VIM, IMP, OXA-48-like, and GES variants [2]. Recent genomic studies indicate that these enzymes show a certain degree of geographic clustering: KPC predominates in reports from China, Brazil, and the US; IMP remains especially important in Japan and East Asia; VIM-1 and OXA-48 have played a major role in Spain; OXA-48-like enzymes are prominent in South Africa and North Africa; and NDM has emerged in both Europe and outbreak settings associated with extremely limited therapeutic options [23,183,184,185,186,187,188,189].

The first carbapenemase linked to *S. marcescens* was SME-1, a chromosomal Ambler class A enzyme [182]. Early reports showed that SME-producing isolates displayed resistance to imipenem with reduced susceptibility to meropenem while often retaining susceptibility to broad-spectrum cephalosporins, a phenotype that remains historically distinctive [181,182]. Although SME enzymes have not become globally dominant, they are important because they demonstrate that *S. marcescens* is not merely a passive recipient of mobile carbapenemase genes but also a species in which carbapenemase evolution has occurred endogenously [2,182].

Among acquired carbapenemases, KPC enzymes are the most prominent globally in *S. marcescens*. Earlier reports identified plasmid-mediated KPC-2 in *S. marcescens* in China during 2006–2007, with evidence of clonal dissemination among hospital isolates [2]. More recently, Jia et al. described two bloodstream isolates from a 36-year-old woman and a 49-year-old man in China, both with bacteremia caused by KPC-2-producing *S. marcescens* [187]. Both isolates carried *bla*_KPC-2_ on IncR plasmids, together with additional resistance determinants; one also harbored *bla*_CTX-M-14_, *qnrS1*, and *aac(6′)-Ic*, illustrating the growing complexity of the *Serratia* spp. resistome [187]. Phenotypically, these isolates were resistant to multiple β-lactams, including carbapenems, while amikacin and tobramycin remained active [187].

In Brazil, KPC appears to be deeply established in nosocomial *S. marcescens*. A large outbreak study conducted during the COVID-19 period in southern Brazil analyzed 170 meropenem-non-susceptible isolates, of which 92.2% carried *bla*_KPC_, whereas *bla*_NDM-1_ was detected in only 3.6% [189]. The outbreak-associated isolates clustered in a single major phylogenetic clade, and *bla*_KPC_ was located on an IncP6 plasmid, consistent with clonal expansion combined with plasmid-mediated dissemination [189]. In the same broad epidemiologic context, an ICU outbreak study from Brazil also confirmed clonal spread of MDR carbapenemase-producing *S. marcescens* and reinforced the role of KPC in national hospital outbreaks [23].

KPC-producing *S. marcescens* has also been described in the US. In Florida, a KPC-3-producing outbreak involved 14 patients across acute-care hospitals and a long-term care facility [185]. All patients were linked epidemiologically to the same neighboring long-term care facility, indicating inter-facility transmission rather than isolated sporadic emergence [185]. Clinical specimens were predominantly respiratory, although BSIs were also documented. Most isolates were highly resistant to carbapenems and cephalosporins, but amikacin and ceftazidime–avibactam retained activity in many cases [185].

In highly vulnerable populations, KPC-producing *S. marcescens* may be particularly devastating. In a Brazilian case series of 11 hematopoietic stem cell transplantation patients colonized or infected with CR *S. marcescens*, KPC was the most prevalent carbapenemase, detected in 8 of 11 patients, and mortality reached 64% [190]. These findings emphasize the clinical burden of KPC-positive *Serratia* spp. in profoundly immunocompromised hosts.

NDM-1 is especially concerning in *S. marcescens* because this species is intrinsically resistant to polymyxins, thereby further narrowing therapeutic options. Gruber et al. described a pan-drug-resistant (PDR) clinical isolate carrying *bla*_NDM-1_ on a 140 kb IncA/C plasmid transferable by conjugation to *E. coli* and *K. pneumoniae* [183]. The isolate was recovered in Germany in a hospital setting and was linked to nosocomial transmission: one strain was detected on rectal screening, and a clonally related isolate was later recovered from the urine of another patient who developed a UTI after occupying the same room [183]. The isolate was non-susceptible to all tested agents, with only intermediate activity of minocycline, illustrating the extreme therapeutic limitations imposed by NDM in *Serratia* spp. [183].

Later surveillance and outbreak studies suggest that NDM has continued to spread, although usually at a lower frequency than KPC in some settings. In the Brazilian COVID-19-era outbreak, *bla*_NDM-1_ accounted for 3.6% of meropenem-non-susceptible isolates, confirming its presence within the same institutional ecosystem dominated by KPC [189]. Environmental genomic work from low- and middle-income countries neonatal wards also identified NDM-1-carrying *S. marcescens* on hospital surfaces, particularly in Bangladesh, where isolates carried *bla*_NDM-1_ on IncF or IncL/M plasmids, underscoring the role of hospital environments as silent reservoirs of carbapenemase-positive *Serratia* spp. [191].

In East Asia, and particularly in Japan, IMP enzymes appear to represent a longstanding carbapenemase lineage in *S. marcescens*. A nationwide Japanese study evaluated 5135 clinical isolates collected between 1994 and 2016 and found that 27 isolates (0.53%) exhibited resistance to ertapenem and/or meropenem, 10 were phenotypically confirmed as carbapenemase producers [188]. WGS identified *bla*_IMP_ in eight isolates, including seven *bla*_IMP-1_ and one *bla*_IMP-11_, the latter representing the first report of IMP-11 in *S. marcescens* [188]. These isolates were MDR, showing broad resistance to β-lactams, fluoroquinolones, tigecycline, minocycline, and trimethoprim–sulfamethoxazole [188].

This Japanese study also provides important historical context, noting that IMP-1 was first identified in 1991 in Japan in a clinical *S. marcescens* isolate, embedded in a class 1 integron [188]. Phylogenomic comparison with international genomes suggested genetic relatedness between Japanese isolates and strains from abroad, raising the possibility of cross-border dissemination, although firm conclusions about imported cases were limited by the lack of travel history data [188]. Thus, IMP-producing *S. marcescens* in Japan appears to reflect both long-term endemic circulation and potential international linkage.

VIM enzymes, particularly VIM-1, have played a major role in European CR *S. marcescens*. In Madrid, Spain, Pérez-Viso et al. analyzed 35 patient-level carbapenemase-producing isolates recovered between 2016 and 2018, of which 29 produced VIM-1 and six produced OXA-48 [186]. These isolates were recovered from both ICU and non-ICU settings, with rectal and respiratory samples being the most common sources [186]. Molecular analysis showed that almost all isolates carried a shared IncL plasmid backbone; in the VIM-producing strains, this plasmid harbored a class 1 integron containing *bla*_VIM-1_ together with *aacA4*, *dfrB1*, *aadA1*, *catB2*, *qacE*Δ1, and *sul1*, demonstrating how integrons and plasmids jointly drive dissemination of multidrug resistance [186].

Austria provides an example of severe infection caused by VIM-positive *S. marcescens*. Lepuschitz et al. reported an MDR carbapenemase-producing isolate recovered from bronchoalveolar lavage of a 68-year-old man with chronic obstructive pulmonary disease and multiple severe comorbidities [184]. WGS analysis identified *bla*_VIM-1_ together with *bla*_ACC-1_, *bla*_SRT-2_, and additional genes conferring resistance to aminoglycosides, quinolones, tetracyclines, and sulfonamides. IncHI2 and IncHI2A plasmids were also present [184]. The isolate was resistant to nearly all tested β-lactams and fluoroquinolones, whereas amikacin and gentamicin remained susceptible [184].

OXA-48-like enzymes are increasingly recognized in *S. marcescens*. In the Madrid hospital cohort, six isolates produced OXA-48, and their plasmids were closely related to the globally disseminated IncL-pOXA-48a-like backbone, supporting the view that *S. marcescens* participates in the same epidemic plasmid networks previously associated mainly with *K. pneumoniae* and *E. coli* [186].

In South Africa, OXA-48-like carbapenemases appear particularly important. Overmeyer et al. studied CR *S. marcescens* from a tertiary hospital over 2015–2020 and found that *bla*_OXA-48-like_ was the most common carbapenemase, present in 86% (18/21) of sequenced CR isolates [192]. Although several phylogenetic clusters were observed, no single dominant clone accounted for all cases, suggesting that both clonal spread and plasmid dissemination contributed to the local epidemiology [192].

North Africa has also emerged as an important region for OXA-48-like *Serratia* spp. In Tunisia, the first OXA-181-producing *S. marcescens* was reported between 2017 and 2019 [193]. Four temocillin-resistant isolates were recovered, mostly from young patients in cardiology and orthopedic wards; two produced OXA-48, and two produced OXA-181 [193]. The OXA-181-producing isolates showed reduced susceptibility rather than high-level resistance, with low carbapenem MICs, illustrating how OXA-48-like enzymes may be difficult to infer from phenotype alone [193]. Importantly, *bla*_OXA-181_ was located together with *qnrS1* on an IncX3/ColKP3 plasmid highly similar to a plasmid previously described in the United Arab Emirates, providing strong evidence for international plasmid circulation and probable cross-border dissemination [193].

Although less frequent than KPC, NDM, IMP, VIM, or OXA-48-like enzymes, GES variants with carbapenemase activity are clearly part of the *S. marcescens* carbapenemase landscape. In Japan, Nakanishi et al. described an ICU outbreak caused by GES-5-producing *S. marcescens* involving six CR strains isolated from three ICU patients between May and October 2020 [194]. All isolates were resistant to imipenem, meropenem, and ceftazidime. PCR screening for *bla*_IMP_, *bla*_NDM_, *bla*_KPC_, and *bla*_OXA-48-like_ was negative, but *bla*_GES_ was detected, and WGS identified a novel plasmid carrying *bla*_GES-5_ [194]. One related isolate lacking *bla*_GES-5_ showed significantly lower MICs, strongly supporting a direct contribution of GES-5 to carbapenem resistance in this outbreak lineage [194].

Brazil has also contributed to the diversification of GES-producing *Serratia* spp. Streling et al. described a new GES-type carbapenemase, GES-16, in two CR clinical isolates recovered in Rio de Janeiro in 2005, one from blood and one from the lower respiratory tract, both from ICU patients [195]. The isolates were clonally related and carried *bla*_GES-16_ together with *bla*_OXA-10_. The *bla*_GES-16_ gene was located in a defective class 1 integron on a 30 kb non-conjugative plasmid, followed by *dfr22*, *aac(6′)-IIc*, and *aadA1* [195]. Biochemical analysis demonstrated carbapenemase activity, with imipenem being the carbapenem most efficiently hydrolyzed [195]. These findings highlight the ongoing evolution of GES-type enzymes in *S. marcescens*.

Across carbapenemase classes, several patterns recur. First, the intrinsic resistance of *S. marcescens* to polymyxins renders carbapenemase acquisition particularly dangerous because it eliminates one of the agents often reserved for treatment of CR Gram-negative infections [2,183]. Second, KPC-producing isolates may retain susceptibility to ceftazidime–avibactam and meropenem–vaborbactam, whereas metallo-β-lactamase producers such as NDM, VIM, and IMP are associated with more restricted β-lactam treatment options [183,185,189]. Third, carbapenemase-positive *S. marcescens* may not always display uniformly high carbapenem MICs, especially with OXA-48-like enzymes, making molecular detection essential for accurate identification [186,193].

**Figure 5:**
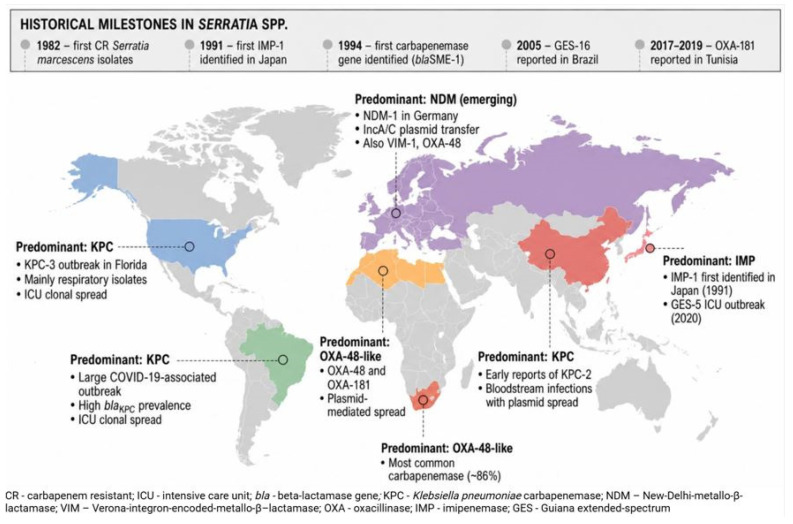
Global epidemiology and geographic distribution of carbapenemases in *Serratia* spp. (Figure created in https://imgcreatorai.io/, accessed on 1 May 2026).

Overall, the epidemiology of carbapenemase-producing *S. marcescens* reflects a combination of clonal expansion, plasmid dissemination, integron-mediated gene capture, and inter-facility as well as cross-border transmission (Figure 5). At the patient level, these organisms are repeatedly associated with ICUs, long-term care facilities, hematology/transplant units, and severely ill or device-dependent patients, and they are recovered from a wide range of specimens, including blood, respiratory samples, urine, rectal surveillance swabs, and bronchoalveolar lavage [23,183,184,185,186,187,188,189,190,192,193,194,195]. The recurring detection of plasmid types such as IncR, IncP6, IncA/C, IncL, IncX3/ColKP3, IncHI2, and IncHI2A underscores the central role of mobile genetic elements in shaping the global carbapenemase ecology of *Serratia* spp. [183,184,186,187,188,189,193].

### 6.3. Non-Carbapenemase-Mediated Carbapenem Resistance

Carbapenem resistance in *S. marcescens* is not exclusively mediated by carbapenemase production. An alternative and clinically relevant mechanism involves the combination of reduced outer membrane permeability due to porin loss and the co-expression of β-lactamases, most commonly AmpC or ESBLs. In this setting, decreased influx of carbapenems into the periplasmic space enhances the relative impact of enzymes that, on their own, generally have limited hydrolytic activity against carbapenems. As a result, clinically relevant carbapenem non-susceptibility or resistance may emerge even in the absence of a bona fide carbapenemase [157,192].

*S. marcescens* intrinsically produces a chromosomally encoded AmpC β-lactamase, which, when overexpressed or derepressed, contributes significantly to β-lactam resistance [151]. However, AmpC activity alone is generally insufficient to confer high-level resistance to carbapenems. A critical additional factor is the reduction or loss of OMPs, which limits antibiotic penetration into the bacterial cell. Under these conditions, even β-lactamases with limited activity against carbapenems may contribute to clinically relevant resistance.

Several studies have documented this mechanism in clinical isolates of *S. marcescens*, particularly in nosocomial settings. CR isolates lacking carbapenemase genes were frequently found to exhibit a combination of ESBL or AmpC production together with alterations in OMPs [166,168,196]. Loss or modification of major porins, analogous to OmpF and OmpC in other *Enterobacterales*, reduces intracellular antibiotic concentrations and enhances the impact of β-lactamase activity.

The strongest experimental evidence for this mechanism in *S. marcescens* comes from a nosocomial outbreak study in which meropenem resistance was shown to be comediated by chromosomal AmpC β-lactamase overproduction and OMP loss [157]. This study clearly demonstrated that carbapenem resistance can arise in the absence of carbapenemase genes when permeability defects are combined with increased β-lactamase expression.

More recent genomic data further support this mechanism. In a study from South Africa, CR *S. marcescens* isolates lacking identifiable carbapenemase genes were found to harbor combinations of β-lactamases such as *bla*_SRT-1_, *bla*_OXA-1_, and *bla*_CTX-M_, together with phenotypic carbapenem resistance, suggesting a role for ESBL/AmpC activity combined with reduced permeability [192]. These findings highlight that carbapenem resistance may occur even when molecular carbapenemase testing is negative.

This mechanism is consistent with broader observations in *Enterobacterales*, where ESBL or AmpC hyperproduction combined with decreased outer membrane permeability represents a recognized pathway to carbapenem resistance [197]. Although less common than carbapenemase-mediated resistance, this mechanism remains clinically relevant, particularly in environments with high antibiotic selective pressure.

Recent reviews confirm that the combination of porin loss and β-lactamase overexpression continues to play an important role in *S. marcescens*, even in the era of widespread carbapenemases [1,82]. In particular, reduced expression of OmpF-like porins together with AmpC overproduction has been shown to contribute to decreased carbapenem susceptibility.

From a clinical perspective, recognition of this mechanism is essential. These isolates often display borderline or variable carbapenem MICs and may not be detected by routine carbapenemase-focused diagnostics. Therefore, carbapenem-non-susceptible but carbapenemase-negative isolates should prompt consideration of combined AmpC/ESBL activity and reduced permeability. This mechanism may also represent an intermediate evolutionary step toward more stable high-level resistance under sustained antibiotic pressure.

### 6.4. Other Resistance Mechanisms (Aminoglycosides, Fluoroquinolones)

Resistance to aminoglycosides in *S. marcescens* is mediated by multiple mechanisms, among which aminoglycoside-modifying enzymes (AMEs) represent the most prevalent. These enzymes inactivate aminoglycosides through acetylation (AAC), phosphorylation (APH), or adenylation (ANT), thereby reducing their affinity for the bacterial 30S ribosomal subunit and impairing antimicrobial activity [198,199]. Genes encoding AMEs are frequently located on mobile genetic elements, including plasmids, transposons, and integrons, which facilitate horizontal gene transfer and contribute to the rapid dissemination of resistance determinants [200].

In *S. marcescens*, commonly identified AMEs include AAC(6′)-I, ANT(2″), and APH variants, often coexisting within the same isolate and conferring resistance to multiple clinically relevant aminoglycosides such as gentamicin, tobramycin, and amikacin [105,201]. Additionally, *S. marcescens* harbors a chromosomally encoded AAC(6′)-Ic enzyme, which is typically expressed at low levels and does not confer intrinsic resistance. However, exposure to aminoglycosides may select for hyperproducing mutants, leading to clinically significant resistance [105].

A particularly concerning mechanism is target modification via 16S rRNA methylation, which results in high-level resistance to nearly all clinically relevant aminoglycosides. Plasmid-mediated methyltransferases such as ArmA, RmtA, RmtB, and RmtC have been increasingly reported in *Enterobacterales*, including *S. marcescens*, and are frequently associated with MDR phenotypes and co-carriage of other resistance genes [202,203]. Additional mechanisms, including reduced outer membrane permeability and efflux pump activity, may contribute to resistance but generally play a secondary role compared to enzymatic modification [50].

Epidemiological data indicate that aminoglycoside resistance in *S. marcescens* is heterogeneous and strongly influenced by local epidemiology. Early surveillance studies demonstrated that 19.2% of aminoglycoside-resistant Gram-negative isolates in the US were *Serratia* spp., with a high prevalence of AMEs such as AAC(6′) and ANT(2″). Similarly, data from Asia reported that 42.7% of aminoglycoside-resistant Gram-negative isolates were *Serratia* spp., often harboring multiple resistance determinants [204].

More recent surveillance studies suggest that resistance rates remain moderate but clinically relevant. In a multicenter study from US hospitals (2002–2004), 7.1% of *S. marcescens* isolates were resistant to tobramycin and 0.8% to amikacin, with additional isolates showing intermediate susceptibility [205]. Comparable findings from South Korea demonstrated 7.5% resistance to amikacin, with resistant strains frequently carrying ArmA methylase and AAC(6′)-Ib enzymes, highlighting the importance of plasmid-mediated resistance [206].

Global surveillance programs provide further insight into prevalence trends. Data from the SENTRY Antimicrobial Surveillance Program indicate that aminoglycosides, particularly amikacin, retain relatively good activity against *S. marcescens*, although resistance rates typically range between 5% and 15% depending on geographic region and study period [207,208]. Nevertheless, the emergence of 16S rRNA methylases has significantly impacted resistance epidemiology, as these enzymes confer high-level resistance to all aminoglycosides and are increasingly detected in MDR and carbapenemase-producing strains [202].

Importantly, resistance rates may be substantially higher in hospital outbreak settings. Numerous nosocomial outbreaks involving aminoglycoside-resistant *S. marcescens* have been reported, particularly in ICUs and immunocompromised patients, where clonal dissemination and horizontal gene transfer contribute to elevated local prevalence [2,105].

Fluoroquinolones exert their antibacterial activity by targeting DNA gyrase and topoisomerase IV, enzymes essential for DNA replication and transcription. DNA gyrase, encoded by the *gyrA* and *gyrB* genes, is the primary target in Gram-negative bacteria, including *Serratia* spp. [209]. Historically, *S. marcescens* has been considered highly susceptible to fluoroquinolones, with early studies reporting near-universal susceptibility among clinical isolates [201,210].

However, temporal trends indicate a gradual decline in susceptibility. Surveillance data from Taiwan demonstrated a decrease in ciprofloxacin susceptibility from 99% in 1985–1986 to 80% in 1996–1997 among *S. marcescens* isolates [211]. Global surveillance programs indicate that fluoroquinolone resistance in *S. marcescens* generally remains below 10–20%, although significant regional variation exists, and higher rates may occur in nosocomial settings [207,212].

Resistance to fluoroquinolones in *S. marcescens* arises through multiple mechanisms, often acting in combination. The most important mechanism involves mutations in the quinolone resistance-determining region (QRDR) of the *gyrA* gene. Single amino acid substitutions in GyrA significantly reduce fluoroquinolone binding and have been consistently associated with resistance in clinical isolates [213,214]. Experimental studies have shown that spontaneous ciprofloxacin-resistant mutants can emerge rapidly under selective pressure due to *gyrA* mutations [215].

Alterations in outer membrane permeability also contribute to resistance. The Omp1 porin has been identified as a major entry pathway for ciprofloxacin in *S. marcescens*, and loss or reduced expression of this porin leads to increased MICs not only for fluoroquinolones but also for β-lactams and aminoglycosides [216,217].

Efflux pumps play a critical role, particularly those belonging to the RND family. Among these, SdeAB is the primary efflux system mediating resistance to fluoroquinolones such as ciprofloxacin, norfloxacin, and ofloxacin [218,219]. Additional efflux systems, including SdeXY and SdeCDE, exhibit substrate specificity, while the SmdAB pump, a member of the ABC family, has also been shown to increase MICs for multiple quinolones [220]. Notably, environmental exposure to disinfectants such as cetylpyridinium chloride may select for mutations that upregulate efflux activity and confer cross-resistance to antibiotics [221].

Plasmid-mediated resistance mechanisms further contribute to the spread of fluoroquinolone resistance. The *qnr* gene family (*qnrA*, *qnrB*, *qnrS*, *qnrC*, *qnrD*) encodes pentapeptide repeat proteins that protect DNA gyrase and topoisomerase IV from quinolone inhibition, typically conferring low-level resistance [222]. Although the prevalence of *qnr* genes in *S. marcescens* appears relatively low, they have been detected in 2.4% of clinical isolates in one study from South Korea [196]. Importantly, the presence of *qnr* genes facilitates the selection of higher-level resistance through additional chromosomal mutations.

A chromosomally encoded *qnr*-like determinant, Smaqnr, has also been described in *S. marcescens*, showing approximately 80% homology to *qnrB1* and contributing to reduced susceptibility to ciprofloxacin [223]. This determinant has been identified in multiple clinical isolates, suggesting it may be more widespread than initially recognized.

Another plasmid-mediated mechanism involves the AAC(6′)-Ib-cr enzyme, a variant of an AME capable of acetylating certain fluoroquinolones, including ciprofloxacin, thereby conferring low-level resistance [224]. This mechanism often coexists with *qnr* genes and has been shown to have an additive effect on resistance levels [175].

An overview of the principal determinants contributing to antimicrobial resistance in *Serratia* spp. is provided in Table 2.

## 7. Novel Therapeutic Options and Challenges in the Treatment of *Serratia* spp. Infections

The emergence and global dissemination of MDR and CR *Serratia* spp. have significantly limited available therapeutic options, necessitating the development of novel antimicrobial strategies. Among these, β-lactam/β-lactamase inhibitor combinations (BLICs) and innovative cephalosporins represent a cornerstone in the modern management of infections caused by resistant *Enterobacterales*, including *Serratia* spp. The efficacy of these agents is closely linked to the underlying resistance mechanisms, particularly the production of AmpC β-lactamases and acquired carbapenemases such as KPC, OXA-48, and metallo-β-lactamases (MBLs) [225,226].

Ceftazidime–avibactam is one of the most extensively studied BLICs in the context of *Serratia* spp. infections. Avibactam, a diazabicyclooctane inhibitor, effectively inhibits Ambler class A and class C β-lactamases, including chromosomal AmpC enzymes that are intrinsic to *Serratia* spp., as well as selected class D carbapenemases [225,227]. Large-scale surveillance data support the promising in vitro activity of ceftazidime–avibactam against *S. marcescens*. In the ATLAS program, this combination demonstrated high susceptibility rates (97.5%) among clinical *S. marcescens* isolates, with low MIC_50_/_90_ values (0.12/4 and 1/4 mg/L), indicating strong intrinsic activity across diverse infection sources [225]. Similarly, multicenter studies from the US reported susceptibility rates exceeding 99% among AmpC-producing *Enterobacterales*, including *Serratia* spp., even among ceftazidime-non-susceptible isolates [227]. These findings confirm that avibactam effectively restores ceftazidime activity against AmpC-hyperproducing strains, a key resistance mechanism in *Serratia* spp. Real-world clinical experience further highlights both the therapeutic potential and practical challenges associated with the use of ceftazidime–avibactam in *S. marcescens* infections. A reported case of persistent CR *S. marcescens* bacteremia in a critically ill pediatric patient demonstrated successful clinical and microbiological resolution following initiation of ceftazidime–avibactam after failure of multiple conventional and combination antimicrobial regimens. The isolate harbored an OXA-48 carbapenemase and exhibited high-level resistance to carbapenems, with persistent bacteremia despite prolonged therapy with meropenem, aminoglycosides, tigecycline, and other agents. Following confirmation of susceptibility, ceftazidime–avibactam-based therapy resulted in clearance of bloodstream infection and sustained clinical recovery [228]. Further, a case of KPC-producing *S. marcescens* endocarditis was successfully treated with ceftazidime–avibactam combined with surgical intervention, resulting in sustained microbiological clearance and a favorable outcome. Despite the severity of infection, prolonged therapy was well tolerated, with no relapse observed [229]. This case supports the role of ceftazidime–avibactam as a viable option for severe MDR *Serratia* spp. infections and highlights the importance of combined medical and surgical management. However, the activity of ceftazidime–avibactam is significantly compromised in CR isolates producing MBLs. In such cases, susceptibility may decrease dramatically, as illustrated by low activity in imipenem-resistant isolates [225]. This limitation reflects the inability of avibactam to inhibit MBL enzymes, highlighting the critical importance of resistance mechanism-guided therapy. Moreover, while ceftazidime–avibactam demonstrates high in vitro activity against *S. marcescens*, emerging evidence highlights the risk of treatment-emergent resistance. A recent study reported the rapid development of resistance during therapy in a KPC-2-producing strain, driven by a 45-nucleotide duplication in the *bla*_KPC-2_ gene, resulting in the *bla*_KPC-44_ variant. This mutation conferred resistance not only to ceftazidime–avibactam but also to other BLICs, including meropenem–vaborbactam and imipenem–relebactam, while susceptibility to cefiderocol was preserved. These findings emphasize the adaptive potential of *S. marcescens* under antibiotic pressure and the need for mechanism-guided therapeutic strategies [230].

Imipenem–relebactam represents another important BLIC targeting class A and class C β-lactamases. Relebactam enhances imipenem activity by inhibiting KPC and AmpC enzymes, mechanisms frequently implicated in *Serratia* spp. resistance [231]. In vitro studies demonstrate that the addition of relebactam significantly reduces imipenem MIC values in CR *Enterobacterales*, restoring susceptibility in approximately 88% of isolates [232]. However, species-specific differences are notable. *S. marcescens* exhibits higher MIC values and lower susceptibility rates compared to other *Enterobacterales*, with only approximately 67% of isolates achieving susceptibility thresholds. Importantly, reduced susceptibility has been associated with chromosomal factors such as porin loss or mutation, even in the absence of acquired carbapenemases [232]. This highlights the complex interplay between enzymatic resistance and membrane permeability in *Serratia* spp., which may limit the effectiveness of this combination.

Meropenem–vaborbactam is a carbapenem combined with a boronic acid β-lactamase inhibitor with potent activity against KPC-producing *Enterobacterales*. Although data specific to *Serratia* spp. are limited, clinical evidence suggests its efficacy in severe infections caused by CR strains. In a reported case of CR *S. marcescens* bacteremia in a critically ill patient, initial therapy with ceftazidime–avibactam, despite in vitro susceptibility, failed to achieve microbiological clearance, with persistent bacteremia observed during treatment. Following a switch to meropenem–vaborbactam, combined with appropriate source control, rapid clinical improvement was achieved, including resolution of fever, clearance of BSIs, and sustained clinical recovery without recurrence [233]. These findings highlight several important considerations: first, that in vitro susceptibility does not always predict clinical success; second, that treatment failure with ceftazidime–avibactam may occur even in susceptible isolates; and third, that meropenem–vaborbactam may represent a valuable salvage therapy in such cases. However, similar to other novel BLICs, its activity is largely restricted to serine carbapenemase producers and is ineffective against MBL-producing strains [226].

Additional limitations of novel BLICs are evident in infections caused by SME-producing *S. marcescens*, a rare but clinically significant carbapenemase-producing phenotype. In vitro data demonstrate that isolates harboring SME enzymes exhibit resistance to carbapenems, including imipenem and meropenem, and notably, the addition of relebactam does not restore imipenem activity or achieve bactericidal effects. In contrast, meropenem–vaborbactam shows significantly enhanced activity, with marked reductions in MIC values and consistent bactericidal and synergistic effects across tested isolates [234]. These findings suggest that vaborbactam possesses superior inhibitory activity against SME enzymes compared to relebactam, likely due to stronger binding affinity and prolonged enzyme interaction. Importantly, despite baseline susceptibility to extended-spectrum cephalosporins such as ceftazidime, resistance development during therapy has been reported in SME- and AmpC-producing strains, raising concerns regarding therapeutic durability [234]. Collectively, these data highlight the heterogeneity of resistance mechanisms in *S. marcescens* and demonstrate that not all novel BLICs are equally effective, underscoring the need for precise, mechanism-guided antimicrobial selection in clinical practice.

Aztreonam–avibactam is an emerging combination specifically designed to overcome MBL-mediated resistance. Aztreonam is inherently stable against MBLs, while avibactam inhibits co-produced serine β-lactamases (ESBLs, AmpC, KPC, OXA-48), thereby restoring aztreonam activity [226,235]. Recent large-scale surveillance studies demonstrate near-complete activity of aztreonam–avibactam against *Enterobacterales*, including CR isolates, with >99.9% susceptibility rates [226]. Importantly, this combination retains activity against isolates resistant to both ceftazidime–avibactam and meropenem–vaborbactam [235]. Given the increasing global prevalence of MBL-producing strains, aztreonam–avibactam is expected to play a critical role in the future management of difficult-to-treat *Serratia* spp. infections.

Cefiderocol, a siderophore cephalosporin, represents a novel class of antibiotics utilizing iron transport systems to facilitate active bacterial uptake. This “Trojan horse” mechanism enables efficient penetration through the outer membrane of Gram-negative bacteria, overcoming permeability-related resistance mechanisms [236]. In vitro studies have demonstrated potent activity of cefiderocol (S-649266) against *Enterobacterales*, including carbapenemase-producing strains such as KPC and NDM producers, with MIC_90_ values ≤ 1 mg/L across multiple species, including *S. marcescens* [236]. Notably, cefiderocol retains activity against both serine and MBL producers, distinguishing it from most novel BLICs. However, combination studies suggest that synergy may vary depending on the organism, with cefiderocol-based combinations showing limited synergistic effects against *S. marcescens* compared to other pathogens.

Recent environmental surveillance studies further emphasize the growing complexity of AMR in *Serratia* spp. and related *Enterobacterales*, particularly in the context of last-line agents such as cefiderocol. Analysis of hospital wastewater isolates revealed a high prevalence of cefiderocol-resistant strains, including *S. marcescens*, many of which exhibited MDR, extensively drug-resistant, or even PDR phenotypes. Notably, these isolates frequently harbored multiple β-lactamase and carbapenemase genes, often in combination (e.g., KPC, VIM, OXA-type enzymes), contributing to elevated MIC values and reduced susceptibility to multiple antibiotic classes. Despite this extensive resistance, susceptibility to newer agents such as aztreonam–avibactam was consistently preserved, while lower resistance rates were observed for meropenem–vaborbactam and imipenem–relebactam [237]. These findings highlight the remarkable genetic plasticity and adaptive capacity of *S. marcescens*, driven by plasmid-mediated gene transfer and environmental selective pressures. Importantly, the study underscores the role of hospital wastewater as a reservoir and dissemination pathway for highly resistant *Serratia* spp. strains, reinforcing the need for integrated surveillance strategies and the continued development of effective therapeutic options.

WGS studies provide critical insights into the molecular basis of resistance in *S. marcescens*. MDR strains frequently harbor multiple resistance genes, including *bla*_KPC-2_, *bla*_SRT-1_, and *bla*_CTX-M-3_, conferring resistance to a wide range of antimicrobial classes [238]. These resistance determinants are often located on mobile genetic elements, including IncX and IncL/M plasmids, facilitating horizontal gene transfer and rapid dissemination across bacterial populations. Phylogenetic analyses reveal close genetic relationships between clinical isolates and globally distributed strains, suggesting both nosocomial transmission and international spread.

Finally, the expanding arsenal of novel antimicrobials has significantly improved treatment options for MDR *Serratia* spp. Nevertheless, therapeutic success remains highly dependent on accurate identification of resistance mechanisms. Ceftazidime–avibactam, imipenem–relebactam, and meropenem–vaborbactam are highly effective against AmpC-, KPC- and OXA-48-producing strains, which are common in *Serratia* spp. In contrast, infections caused by MBL-producing strains require alternative strategies, with aztreonam–avibactam and cefiderocol representing the most promising options. The increasing complexity of resistance mechanisms, including porin mutations and combined enzymatic pathways, further underscores the need for molecular diagnostics and individualized therapy. Consequently, the selection of appropriate antimicrobial therapy for *Serratia* spp. infections should be guided by both phenotypic susceptibility testing and genotypic characterization of resistance determinants.

**Figure 6:**
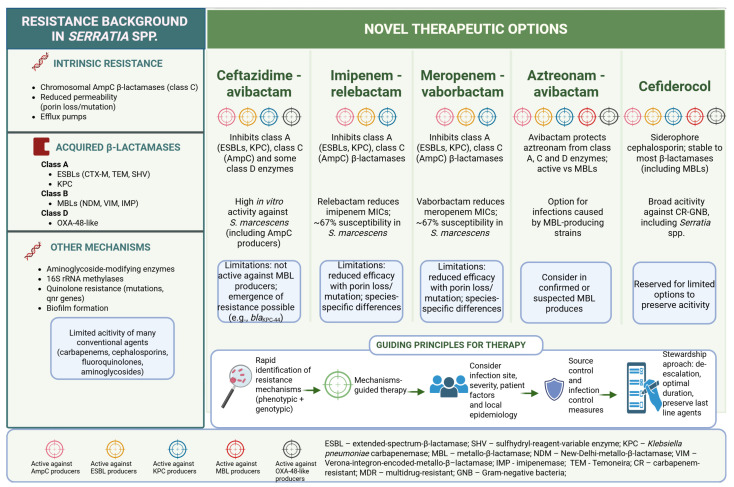
Mechanism-guided therapeutic approach to multidrug-resistant and carbapenem-resistant *Serratia* spp. infections. (Figure created in https://BioRender.com, accessed on 1 May 2026).

The relationship between resistance mechanisms and currently available mechanism-guided therapeutic options for MDR and CR *Serratia* spp. is summarized in Figure 6.

From an antimicrobial stewardship perspective, the management of MDR and CR *Serratia* spp. infections should rely on early optimization of therapy rather than empirical escalation alone. Given the heterogeneity of resistance mechanisms in this genus, treatment decisions should integrate infection severity, infection source, local epidemiology, phenotypic antimicrobial susceptibility testing, and, whenever available, molecular detection of β-lactamases and carbapenemases. Rapid identification of resistance determinants may help distinguish isolates likely to respond to newer BLICs from those requiring alternative approaches, such as aztreonam–avibactam or cefiderocol in the setting of MBL production. In addition, appropriate source control, avoidance of unnecessary broad-spectrum exposure, and de-escalation based on microbiological results remain essential components of therapy. Therefore, stewardship-guided management of *Serratia* spp. infections should combine timely, effective treatment with preservation of last-line agents and prevention of further resistance emergence.

## 8. Alternative Therapeutic Options

When conventional and carbapenem-based regimens fail, the selection of a last-line therapy for MDR *Serratia* spp. infections becomes critically constrained, as few antimicrobial classes retain reliable activity against this pathogen. Fosfomycin and tigecycline have emerged as the most frequently considered alternative agents, yet their utility differs substantially. Fosfomycin remains active against a significant proportion of MDR isolates, including strains resistant to carbapenems, while tigecycline activity against *Serratia* spp. is subject to important limitations that must be considered before its use [239,240,241,242].

Tigecycline is licensed for complicated intra-abdominal infections and complicated skin and soft-tissue infections and may be considered for these indications when optimal dosing is used, but it is not recommended for UTIs, BSIs, or hospital-acquired pneumonia [2,243,244]. According to the European Committee on Antimicrobial Susceptibility Testing (EUCAST), tigecycline has insufficient activity against *Serratia* spp., and susceptibility test results should be reported as resistant, irrespective of the actual MIC obtained [242,245]. Despite this, some in vitro surveillance data suggest a more variable picture. Datasets from ICU and pneumonia cohorts have reported that a majority of *Serratia* spp. isolates are inhibited at concentrations ≤ 2–4 mg/L [246,247,248,249,250]. However, an important methodological limitation should be noted: most of these studies were conducted over a decade ago and interpreted MIC values according to Clinical and Laboratory Standards Institute (CLSI) criteria, although CLSI does not define clinical breakpoints for tigecycline and instead refers users to those approved by the US Food and Drug Administration (FDA) [251]. The FDA breakpoints, unlike EUCAST, do not carry species-specific restrictions for *Serratia* spp., which may have led to an overestimation of susceptibility rates. In a systematic review, Kelesidis et al. found susceptibility rates exceeding 90% only in studies that included non-MDR isolates, whereas among six studies focusing specifically on MDR *Serratia* spp., susceptibility fell to approximately 78%. Critically, the authors emphasized that small sample sizes preclude any definitive conclusions about the reliability of tigecycline for treating *Serratia* spp. infections [252]. Sari et al. similarly reported low tigecycline resistance (9%) among *S. marcescens* isolates from a pediatric BSI cohort, but no tigecycline-based treatment courses or clinical outcomes were described, limiting the interpretability of this finding [253]. With the aforementioned limitation in mind, the clinical relevance of these observations remains uncertain. Nonetheless, where no alternative agents remain, higher non-licensed dosing regimens (100 mg every 12 h with or without a 200 mg loading dose) may be considered in seriously ill patients, as pharmacokinetic/pharmacodynamic (PK/PD) modeling predicts target attainment against strains with MICs of up to 1 mg/L, although evidence for this approach remains limited and potentially biased [254].

Clinical evidence for tigecycline in confirmed *S. marcescens* infections is confined to case reports, small case series, and heterogeneous observational cohorts, yet these data point to a possible role in selected scenarios. In a case series of hematopoietic stem cell transplant patients with CR *S. marcescens*, tigecycline combined with gentamicin was among the main therapies employed. Overall mortality was 64%, largely attributable to the severity of the underlying condition and the scarcity of available treatment options [190]. More encouraging results have been reported at the individual patient level, when successful treatment of *S. marcescens* ventriculitis (isolate MIC 2 mg/L) was achieved in a pediatric patient. Combined intravenous and intraventricular tigecycline, administered after failure of standard therapy, resulted in clinical recovery and cerebrospinal fluid sterilization [255]. Avcu et al. reported clinical response and microbiological eradication each in 80% of pediatric MDR *Serratia* spp. cases treated with tigecycline-based combination regimens, despite MICs ranging up to 12 mg/L [256]. A favorable outcome was similarly described in peritonitis caused by an extensively resistant MBL-producing *S. marcescens* isolate treated with tigecycline plus moxifloxacin [257].

Analyses focusing specifically on *S. marcescens* infections indicate that tigecycline is used relatively infrequently and primarily as a last-line or combination agent, reflecting both the intrinsic reduction in susceptibility characterized by EUCAST, uncertainty in its efficacy, and concerns related to pharmacokinetic limitations.

Unlike tigecycline, fosfomycin carries no EUCAST intrinsic resistance classification for *Serratia* spp., and demonstrates favorable in vitro activity and potential clinical utility, representing a valuable option against MDR *Serratia* spp. [258]. Across studies, *Serratia* spp. are consistently listed among *Enterobacterales* that are usually susceptible to fosfomycin in vitro, typically with MIC in the range of 0.25–16 mg/L, often falling at the upper end of the *Enterobacterales* MIC distribution [240,258,259,260,261,262,263]. Ramos et al. highlight that, in clinical practice, high-dose intravenous fosfomycin (typically 16–24 g/day) is primarily employed in combination regimens for MDR Gram-negative infections, including urinary, soft tissue, and intra-abdominal infections [260].

There is a notable lack of *Serratia*-specific clinical studies evaluating fosfomycin for UTIs, so current recommendations rely largely on extrapolation from broader *Enterobacterales* UTI data rather than direct evidence. The European Society of Clinical Microbiology and Infectious Diseases (ESCMID) survey shows that, in real-world practice, intravenous fosfomycin is occasionally combined with other agents for CR *Enterobacterales* infections, including complicated UTIs, yet without organism-specific outcome data or strong evidence to guide its use for *Serratia* spp. UTIs [244]. A study by Udayan et al. found that all *Serratia* spp. urinary isolates, including MDR strains, were 100% susceptible to fosfomycin in vitro, supporting fosfomycin as a promising oral option for treating *Serratia*-associated UTIs, although no *Serratia*-specific clinical outcome data were reported [264]. In a large Turkish cohort, all six *Serratia* spp. urinary isolates recovered from community- and hospital-acquired UTI (including any ESBL producers) were 100% susceptible to fosfomycin in vitro, leading the authors to conclude that fosfomycin is a valuable option for UTIs caused by *Serratia* spp. [265].

Available data suggest that fosfomycin has, at best, a narrow and largely urinary role against MDR *Enterobacterales*, but no robust evidence supports its use for systemic *Serratia* spp. infections, especially outside combination regimens [243,244]. Baquero et al. reported a case series of 24 hospitalized children with *S. marcescens* septicemia who were successfully treated with combination regimens including fosfomycin plus carbenicillin or gentamicin, with the latter achieving an 89% cure rate [266]. In a compassionate-use series of 13 severe infections due to PDR Gram-negative bacilli, three *S. marcescens* cases (mostly bloodstream or deep infections) were treated with high-dose intravenous fosfomycin, usually combined with meropenem, contributing to an overall 62% clinical cure rate and frequent in vitro synergy with meropenem, though *Serratia*-specific outcomes were too few for firm conclusions [267]. Rodriguez et al. reported successful treatment of a patient from whom nine MDR, *bla*_KPC-2_ positive *S. marcescens* isolates were recovered from sequential bone and soft-tissue samples, all susceptible in vitro to ceftazidime and fosfomycin, which were administered in combination [268]. In the soft-tissue PK/PD study by Frossard et al., intravenous fosfomycin achieved high interstitial-fluid concentrations in muscle and adipose tissue, and simulated exposure corresponding to a single 4–8 g dose produced complete killing of *S. marcescens* isolates with MICs ≤ 16 mg/L within one dosing interval, suggesting that adequate dosing can eradicate susceptible *Serratia* spp. at the tissue site [269].

Consequently, the successful use of tigecycline and fosfomycin for *Serratia* spp. infections appears to depend on careful patient and infection-site selection, confirmation of in vitro susceptibility, and, in many reported cases, their incorporation into combination regimens rather than use as monotherapy.

## 9. Novel and Adjunctive Therapeutic Strategies

**Figure 7:**
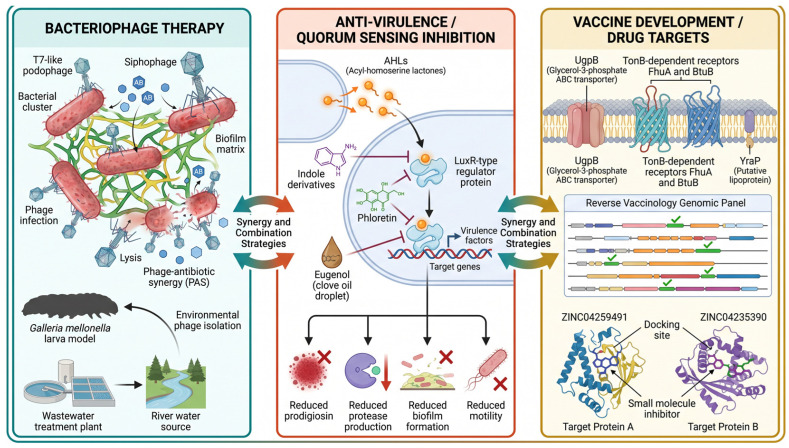
Emerging therapeutic strategies against *S. marcescens*: phage therapy, antivirulence approaches, and vaccines. (Figure created in https://imgcreatorai.io/, accessed on 1 May 2026).

Emerging therapeutic strategies for *Serratia* spp., particularly *S. marcescens*, are increasingly oriented toward adjunctive approaches that target bacterial persistence and virulence rather than viability alone. As a versatile opportunistic pathogen, *S. marcescens* exhibits substantial intrinsic and acquired resistance mechanisms (AmpC, ESBL, carbapenemases, efflux pumps, porin loss), combined with a pronounced capacity for biofilm formation, all of which complicate treatment and highlight the need for adjunctive options (Figure 7) [16].

### 9.1. Bacteriophage Therapy and Phage–Antibiotic Synergy

*Serratia*-specific lytic bacteriophages have demonstrated the ability to disrupt biofilms, while phage–antibiotic synergy (PAS) is emerging as a promising strategy. Duan et al. reported the successful treatment of a 59-year-old man with MDR *S. marcescens* pulmonary infection using a personalized lytic bacteriophage Spe5P4, in combination with amikacin and meropenem after prolonged antibiotic failure. Treatment was associated with significant clinical and radiological improvement, accompanied by a progressive decline and eventual eradication of *S. marcescens* from pleural fluid, while phage titers remained sustained. No treatment-related adverse effects or organ dysfunction were observed, and inflammatory markers decreased during follow-up. Notably, no phage-resistant strains emerged in vivo, nor was there evidence of increased virulence or biofilm formation. Instead, some isolates exhibited increased antibiotic susceptibility [270].

Weber et al. described SALSA (vB_SmaP-SALSA), a strictly lytic T7-like podophage active against selected clinical *S. marcescens* isolates, with a genome lacking virulence, integrase, and lysogeny-associated genes. Although SALSA alone produced rapid initial bacterial killing, regrowth occurred due to phage resistance, and antibiotics such as ampicillin-sulbactam or meropenem alone achieved only transient reductions. In contrast, combining SALSA with either antibiotic, including ampicillin-sulbactam despite intrinsic resistance, resulted in sustained suppression and complete bacterial eradication, even at low phage and antibiotic concentrations. This synergy, dependent on productive phage infection, is likely mediated by phage-induced outer membrane changes that enhance antibiotic uptake, supporting phage–antibiotic combinations as a more effective approach than phage monotherapy for *Serratia* spp. infections [271].

Horton et al. isolated two novel lytic bacteriophages, Sm10b_1 and Sm12, from river water using a bloodstream *S. marcescens* isolate as the host. Both phages demonstrated relatively broad activity by infecting at least 40% of tested clinical and environmental *Serratia* spp. strains. Genomic analysis confirmed that Sm10b_1, a podophage, and Sm12, a siphophage, represent new taxa and lack integrases, virulence factors, and antibiotic resistance genes, supporting their safety for therapeutic consideration [272]. In a *Galleria mellonella* infection model, phage treatment significantly improved survival, with a single dose of Sm10b_1 rescuing a substantial proportion of larvae from otherwise lethal *S. marcescens* infection, and similarly enhancing survival in infections caused by *S. odorifera*. These findings demonstrate that environmentally sourced, broad-host-range lytic phages can provide effective in vivo protection against *Serratia* spp. infections [272].

Another study demonstrated that bacteriophages isolated from wastewater and soil can effectively kill an antibiotic-resistant, biofilm-forming *S. marcescens* strain, supporting their potential therapeutic use. In broth, the wastewater phage (S.wph) rapidly and durably suppressed bacterial growth, clearly outperforming ampicillin, while a combination of ampicillin with the soil phage (S.So.ph) produced additional inhibition, suggesting useful PAS. Both phages, especially when combined, significantly reduced biofilm biomass and exopolysaccharide production, key factors in chronic and device-associated *Serratia* spp. infections, indicating that such phage preparations could improve treatment outcomes where conventional antibiotics alone are insufficient [273].

These findings support bacteriophage therapy, ideally in rational combination with antibiotics, as a feasible and increasingly evidence-based approach for treating challenging *Serratia* spp. infections, while highlighting the need for individualized phage selection, rigorous genomic characterization, and systematic evaluation of PAS. Because phage–antibiotic interactions are highly pair-specific and may be either synergistic or antagonistic, empirical testing of each combination is essential [274].

### 9.2. Quorum-Sensing Inhibition and Antivirulence Strategies

In parallel, antivirulence strategies, especially QS inhibition, represent a promising avenue. *Serratia* spp. use acyl-homoserine lactone (AHL) QS systems (e.g., *smaI*/*smaR*, *swrI*/*swrR*) to regulate biofilm formation and production of virulence factors such as prodigiosin, proteases, lipases, nucleases, hemolysins, and motility. Targeting these systems can attenuate pathogenicity without directly killing bacteria, theoretically reducing selection for resistance. Several works showed that blocking AHL-mediated QS strongly attenuates *S. marcescens* virulence and enhances antibiotic susceptibility without inhibiting growth [16,275].

Marine *Bacillus* spp. SS4 produces a non-bactericidal metabolite that markedly reduces prodigiosin (up to ~87%), protease (~60%), and biofilm thickness while increasing susceptibility to erythromycin and chloramphenicol. It does not degrade AHLs but likely interferes with LuxR-type regulators [276]. A rhizosphere *Bacillus subtilis* R-18 secretes QS-inhibitory compounds whose petroleum ether extract decreases prodigiosin by up to 98%, reduces biofilm biomass 34–68%, and strongly inhibits protease, lipase, hemolysin and biofilm in an MDR urinary isolate, with conserved downregulation of multiple virulence and QS-related genes [277]. Screening 51 indole derivatives identified several (e.g., indole, 6-fluoroindole, and 7-methylindole) that dose-dependently suppress prodigiosin, biofilm, motility, protease and lipase; reduce fimbria-mediated yeast agglutination; deplete extracellular polymeric substance (EPS); and downregulate key QS/biofilm genes (*bsmA*, *fimA*, *pigA*/*pigC*, *smaI*, and *rpoS*), again at concentrations below MIC [278].

Natural small molecules from plants also emerge as promising adjuncts. Phloretin, a phenol from apple peel, disrupts QS in *S. marcescens* NJ01 by markedly lowering C4-HSL and C6-HSL levels; reduces biofilms by ~20–28% alone and by ~50–63% in combination with amikacin, netilmicin, or imipenem; and strongly inhibits multiple virulence traits (protease, prodigiosin, EPS, and motility) [279]. Mechanistically, phloretin induces oxidative stress, alters membrane composition and permeability, causes broad metabolic disturbances, and downregulates genes for adhesins, EPS, protease, pigment and antioxidant defenses, which together increase the susceptibility of biofilm cells to antibiotics [279]. Eugenol, a major clove oil component, shows a similar antivirulence profile in two *S. marcescens* strains. At sub-MIC levels, it reduces biofilms by ~60–75%; disrupts biofilm structure, decreases prodigiosin, hemolysin and protease; impairs swarming and EPS production; and downregulates *flhD*, *fimC*, *bsmA*/*bsmB* and the QS regulator *swrR* [275].

These anti-QS/antivirulence strategies, from indoles and flavonoids to essential-oil components, are framed as adjuvants that weaken biofilms and virulence, making standard antibiotics and host defenses more effective rather than replacing antibiotics. They can be combined with conventional antibiotics to improve the eradication of *Serratia* spp. biofilms and curb MDR infections. Such combination regimens, standard antibiotics plus non-toxic QS inhibitors that reduce virulence and biofilm tolerance, are the most promising therapeutic direction for *Serratia* spp. infections [190,280].

### 9.3. Vaccine Development and Novel Drug Targets

Given the rising incidence of MDR *S. marcescens* infections, vaccines are emerging as a promising therapeutic strategy to prevent disease and reduce reliance on failing antibiotics. Prado et al. in their study used reverse vaccinology and subtractive genomics on 59 *S. marcescens* genomes to identify conserved, non-human-homologous proteins as potential therapeutic targets. The authors predict seven vaccine candidates (including UgpB, TonB-dependent receptors FhuA and BtuB, YraP, two murein/peptidoglycan transglycosylases, and a DUF481 protein) and two drug targets (N(4)-acetylcytidine amidohydrolase and a DUF1428 protein), then use molecular docking and dynamics to propose two small molecules (ZINC04259491 and ZINC04235390) with favorable binding to the drug targets, supporting future vaccine and drug development against MDR *S. marcescens* [281].

## 10. Future Directions and Research Gaps

Despite increasing recognition of *Serratia* spp. as a clinically relevant opportunistic pathogen, significant gaps remain in understanding its epidemiology, resistance, and pathogenic potential. One of the most critical challenges lies in the lack of coordinated and comprehensive surveillance systems. Although local studies have documented rising resistance rates, including a concerning increase in carbapenem resistance, these data are rarely integrated into global frameworks, limiting the ability to track dissemination and anticipate emerging high-risk clones. The underrepresentation of *Serratia* spp. in large-scale surveillance programs further contributes to this gap and underscores the need for genome-based monitoring approaches.

At the same time, therapeutic options remain constrained by the intrinsic and acquired resistance mechanisms of *Serratia* spp., including AmpC β-lactamases and carbapenemases. The gradual loss of efficacy of last-line antibiotics highlights the urgent need for innovative treatment strategies that extend beyond conventional bactericidal approaches. Emerging concepts such as antivirulence therapies, biofilm-targeting agents, and adjunctive compounds show promise, particularly in addressing persistent and device-associated infections, although their clinical relevance remains to be fully established.

Advances in diagnostics represent another key area for improvement. While current technologies enable rapid species identification, the timely detection of resistance mechanisms is still limited, often delaying appropriate therapy. The integration of rapid molecular diagnostics, genomics, and data-driven predictive models may significantly enhance clinical decision-making and infection control, particularly if extended to environmental monitoring in healthcare settings.

Finally, a deeper understanding of *Serratia* spp. virulence and host–pathogen interactions remains essential. Although multiple virulence factors have been described, their regulation and contribution to disease are highly context-dependent and not fully elucidated. The dynamic interplay between bacterial adaptability and host immune response represents a major unresolved aspect of *Serratia* spp. pathogenesis, requiring integrative, multi-omics approaches to clarify mechanisms of persistence, immune evasion, and disease severity.

Taken together, these gaps highlight the need for a multidisciplinary and systems-level approach to *Serratia* spp. research, integrating surveillance, molecular biology, and clinical data to better address the challenges posed by this increasingly important pathogen in the AMR era.

## 11. Conclusions

*Serratia* spp. have emerged as clinically significant opportunistic Gram-negative pathogens with increasing relevance in both community and HAIs. While *S. marcescens* remains the predominant species implicated in human disease, other members of the genus contribute to infection, particularly in immunocompromised and critically ill patients. Their clinical success reflects a high degree of ecological adaptability, underpinned by intrinsic resistance mechanisms, acquisition of mobile genetic elements, and a diverse repertoire of virulence factors that facilitate persistence, transmission, and biofilm formation.

Within healthcare environments, *Serratia* spp. are closely associated with device-related and nosocomial infections, where their ability to survive under adverse conditions and disseminate clonally presents ongoing challenges for infection control. The increasing prevalence of MDR and CR isolates further complicates therapeutic management and highlights the limitations of current antimicrobial strategies.

In parallel, growing research efforts aimed at characterizing the molecular epidemiology and resistance determinants of *Serratia* spp. have revealed considerable heterogeneity among reported findings. However, some inconsistencies among studies remain regarding the prevalence of specific resistance genes, virulence profiles, and susceptibility to certain last-line agents such as tigecycline and cefiderocol. These discrepancies likely reflect geographic variability, differences in surveillance strategies, methodological heterogeneity, and evolving interpretative susceptibility criteria.

The present review provides a comprehensive synthesis of current knowledge regarding the epidemiology, virulence, antimicrobial resistance, and therapeutic strategies associated with *Serratia* spp. By integrating microbiological, molecular, and clinical perspectives, this study highlights the growing public health importance of these pathogens and underscores the urgent need for improved surveillance systems, rapid molecular diagnostics, targeted antimicrobial stewardship, and continued development of innovative therapeutic approaches.

Future progress in the control of resistant *Serratia* spp. will depend heavily on the implementation of coordinated genomic surveillance strategies and the broader application of whole-genome sequencing technologies. WGS-based approaches may substantially improve outbreak detection, tracking of transmission pathways, identification of emerging high-risk clones, and characterization of resistance and virulence determinants across clinical and environmental reservoirs. Importantly, the increasing recognition of hospital wastewater, medical devices, and environmental niches as potential reservoirs of resistant *Serratia* spp. strains highlights the need for integrated surveillance beyond conventional clinical settings.

In this context, adopting a One Health framework is essential for understanding and controlling the dissemination of antimicrobial-resistant *Serratia* spp. The integration of human, environmental, and microbiological data may improve early detection of emerging threats and support more effective prevention and containment strategies. Ultimately, a multidisciplinary and globally coordinated approach combining molecular epidemiology, environmental monitoring, infection control, and antimicrobial stewardship will be critical to reducing the clinical and public health impact of *Serratia* spp. in the era of escalating AMR.

## Figures and Tables

**Table 1:** Major virulence determinants of *Serratia* spp. and their clinical significance.

Virulence Factor/ System	Main Components or Examples in *Serratia* spp.	Biological Role	Contribution to Antimicrobial Resistance and Persistence	References
Flagella and motility	Flagellin, swarming-associated genes	Surface colonization and dissemination	Promotes biofilm maturation and persistence on medical devices	[2,16,21,22,23]
Fimbriae and adhesins	Type 1 fimbriae, curli-like fimbriae, pili-like adhesins	Adhesion to epithelial and abiotic surfaces	Facilitates stable colonization and biofilm initiation	[24,25,26,27,28,29,30]
Capsule	Acidic polysaccharides	Protection from environmental stress and host immunity	Increase tolerance to the host immune mechanisms	[31,32,33,34]
Outer membrane proteins (OMPs)	OmpA-, OmpC-, OmpF-like proteins	Membrane integrity, adhesion, and host interaction	Reduced permeability contributes to multidrug resistance	[35,36,37,38,39,40,41,42,43,44,45,46,47,48,49,50,51,52,53,54,55,56,57,58,59,60,61,62,63,64,65,66,67,68,69,70,71,72,73,74,75,76,77]
Porin regulation and loss	Downregulation or mutation of porins	Decreased uptake of antimicrobial agents	Associated with β-lactam and carbapenem resistance	[36,37,38,39,40,41,42,43,44,45,46,47,48,49,50,51,52,53,54]
Extracellular enzymes	Proteases, lipases, nucleases, hemolysins	Tissue invasion and nutrient acquisition	Enhances dissemination and pathogenicity	[16,17,20,21,22,23,77,78,79,80]
Siderophore	Enterobactin-like systems	Iron scavenging in iron-limited environments	Supports survival during infection	[16,21,23,81]
Type I secretion system (T1SS)	RTX-associated exporters	Secretion of toxins and proteases	Promotes host tissue damage and adaptation	[82,83,84,85,86,87,88]
Type II secretion system (T2SS)	Extracellular enzyme secretion machinery	Export of proteases and lipases	Enhances invasion and environmental persistence	[89,90,91,92,93]
Type III secretion system (T3SS)	Needle-like injectisome	Injection of effector proteins into host cells	Immune modulation and intracellular survival	[16,83,89,94,95,96]
Type V secretion system (T5SS)	Autotransporters	Adhesion and host interaction	Contributes to colonization and persistence	[82]
Type VI secretion system (T6SS)	Contractile secretion apparatus	Interbacterial competition and virulence	Supports niche establishment and survival in polymicrobial environments	[97,98]
Type VIII secretion system (T8SS)	Secretion-associated pathways	Curli-fiber assembly	Promotes adhesion and biofilm formation	[24,25,99,100]
Type X secretion system (T10SS)	Extracellular secretion of large proteins	Controlled cell lysis	Contributes to environmental survival and host interaction	[101,102,103]
Biofilm formation	EPS matrix, extracellular DNA	Protection from environmental stress and host immunity	Increases tolerance to antibiotics and disinfectants	[16,19,21,22,23]
Quorum-sensing systems	LuxIR/SmaIR homologous systems	Regulation of virulence gene expression	Coordinates biofilm formation and stress adaptation	[2,16,19,104]

**Table 2:** Major intrinsic and acquired resistance mechanisms in *Serratia* spp.

Resistance Mechanism	Type	Major Genes/ Mechanisms	Affected Antibiotic Classes	Clinical Implications
AmpC β-lactamase	Intrinsic	*ampC*, AmpR regulatory system	Aminopenicillins, first- and second-generation cephalosporins, cephamycins	Major intrinsic resistance mechanism; may lead to treatment failure during cephalosporin therapy
Reduced outer membrane permeability	Intrinsic/ acquired	Porin loss or downregulation (*OmpF*, *OmpC*-like proteins)	β-lactams, carbapenems	Reduced antibiotic influx; enhances multidrug resistance when combined with β-lactamases
Efflux pump overexpression	Intrinsic/ acquired	RND ^a^-family pumps, MFS ^b^, ABC ^c^ transporters	Fluoroquinolones, tetracyclines, chloramphenicol, β-lactams, aminoglycosides	Contributes to MDR ^d^ phenotype and decreased intracellular drug accumulation
LPS ^e^ modifications	Intrinsic	Constitutive modification of lipid A	Colistin, polymyxins	Intrinsic reduced susceptibility limits the use of colistin
ESBL ^f^ production	Acquired	*bla_CTX-M_*, *bla_TEM_*, *bla_SHV_*	Extended-spectrum cephalosporins, monobactams	Limits use of third-generation cephalosporins; often associated with plasmid-mediated MDR
Class A serine carbapenemase	Acquired	*bla_SME_*, *bla_KPC_*	Penicillins, cephalosporins, carbapenems	Associated with severe healthcare-associated outbreaks and limited therapeutic options
Class B metallo-β-lactamase	Acquired	*bla_NDM_*, *bla_VIM_*, *bla_IMP_*	Almost all β-lactams except aztreonam	Frequently associated with XDR ^g^/PDR ^h^ phenotypes and high mortality
Class D OXA ^i^-carbapenemase	Acquired	*bla* *_OXA-48_*_-like_	Penicillins and carbapenems	May produce low-level carbapenem resistance that complicates laboratory detection
Aminoglycoside-modifying enzymes (AMEs)	Acquired	*aac*, *aph*, *ant*	Aminoglycosides	Reduces the effectiveness of aminoglycosides combination therapy
16S rRNA methylases	Acquired	*armA*, *rmt*	Aminoglycosides	Confers high-level aminoglycoside resistance
Fluoroquinolone target mutations	Acquired	*gyrA*, *gyrB*, *parC*, *parE*	Fluoroquinolones	Reduced fluoroquinolone susceptibility and therapeutic failure
Plasmid-mediated quinolone resistance	Acquired	*qnr* genes, *aac(6′)-Ib-cr*	Fluoroquinolones	Facilitates the emergence of high-level quinolone resistance

^a^ RND-resistance–nodulation–division; ^b^ MFS-major facilitator superfamily; ^c^ ABC-ATP-binding cassette; ^d^ MDR-multidrug resistant; ^e^ LPS-lipopolysaccharide; ^f^ ESBL-extended-spectrum β-lactamases; ^g^ XDR-extensively drug-resistant; ^h^ PDR-pandrug-resistant; ^i^ OXA-oxacillinase.

## Data Availability

No new data were created or analyzed in this study. Data sharing is not applicable to this article.

## Abbreviations

The following abbreviations are used in this manuscript:

ABC	ATP-binding cassette
AHL	Acyl-homoserine lactone
AME	Aminoglycoside-modifying enzymes
AMR	Antimicrobial resistance
BLIC	β-lactamase inhibitor combination
BSI	Bloodstream infection
CLSI	Clinical and Laboratory Standards Institute
CNS	Central nervous system
CR	Carbapenem-resistant
CU	Chaperone–usher
ECDC	European Centre for Disease Prevention and Control
EPS	Extracellular polymeric substance
ESBL	Extended-spectrum β-lactamase
ESCMID	European Society of Clinical Microbiology and Infectious Diseases
EUCAST	European Committee on Antimicrobial Susceptibility Testing
FDA	Food and Drug Administration
HAI	Healthcare-associated infection
ICU	Intensive care unit
LPS	Lipopolysaccharide
MALDI-TOF MS	Matrix-assisted laser desorption/ionization time-of-flight mass spectrometry
MATE	Multidrug and toxic compound extrusion
MBL	Metallo-β-lactamase
MDR	Multidrug resistant
MFS	Major facilitator superfamily
MIC	Minimum inhibitory concentration
MLST	Multilocus sequence typing
NICU	Neonatal intensive care units
OMP	Outer membrane protein
PAS	Phage–antibiotic synergy
PDR	Pan-drug-resistant
PFGE	Pulsed-field gel electrophoresis
PK/PD	Pharmacokinetic/pharmacodynamic
QRDR	Quinolone resistance-determining region
QS	Quorum sensing
RND	Resistance–nodulation–division
SMR	Small multidrug resistance
SNP	Single-nucleotide polymorphism
SSI	Surgical site infections
T10SS	The type X secretion system
T1SS	The type I secretion system
T2SS	The type II secretion system
T3SS	The type III secretion system
T5SS	The type V secretion system
T6SS	The type VI secretion system
T8SS	The type VIII secretion system
US	United States
UTI	Urinary tract infection
WGS	Whole-genome sequencing
XDR	Extensively drug resistant
